# Suspect screening analysis to improve untargeted and targeted UHPLC-qToF approaches: the biodegradability of a proton pump inhibitor medicine and a natural medical device

**DOI:** 10.1038/s41598-023-49948-8

**Published:** 2024-01-02

**Authors:** Luisa Mattoli, Giacomo Proietti, Giada Fodaroni, Claudio Marzio Quintiero, Michela Burico, Mattia Gianni, Emiliano Giovagnoni, Valentino Mercati, Claudio Santi

**Affiliations:** 1 Metabolomics and Analytical Sciences, Aboca SpA, Sansepolcro, AR Italy; 2 Group of Catalysis, Synthesis and Organic Green Chemistry, Department of Pharmaceutical Sciences, University of Perugia, Via del Liceo 1, 06123 Perugia, Italy; 3 Centro di Eccellenza Materiali Innovativi Nanostrutturati (CEMIN), University of Perugia, Via Elce di Sotto 8, 06123 Perugia, Italy

**Keywords:** Environmental chemistry, Environmental impact

## Abstract

Suspect screening and untargeted analysis using UHPLC-qToF are two advanced analytical approaches now used to achieve an extensive chemical profile of samples, which are then typically confirmed through targeted analysis. These techniques can detect a large number of chemical features simultaneously and are currently being introduced into the study of contaminants of emerging concern (CECs) and into the study of the extent of human chemical exposure (the exposome). Here is described the use of these techniques to characterize chemical mixtures derived from the OECD 301F ready biodegradability test (RBT) of a chemical and natural formulation currently used to treat reflux disease and functional dyspepsia. Untargeted analysis clearly evidenced a different behavior between formulations containing only natural products with respect to that containing synthetic and non-naturally occurring substances. Suspect screening analysis improved the untargeted analysis of the omeprazole-based medicine, leading to the tentative identification of a number of omeprazole-derived transformation products, thereby enabling their preliminary quali-quantitative evaluation. Targeted analysis was then performed to confirm the preliminary data gained from the suspect screening approach. The validation of the analytical method for the quantitative determination of omeprazole and its major metabolite, omeprazole sulphide, has provided robust data to evaluate the behavior of omeprazole during the OECD 301F test. Using advanced analytical approaches, the RBT performed on the two products under investigation confirmed that omeprazole is not readily biodegradable, while the medical device made of natural substances has proven to be readily biodegradable.

## Introduction

There are currently several European regulatory frameworks established to protect the environment and human health from the risks and hazards posed by the production of hundreds of thousands of chemicals, e.g., the regulation for registration, evaluation, authorization and restriction of chemicals (REACH, Reg. 2006/1907)^1^, the regulation on Biocides (BPR, Reg. 2012/528)^2^, the directive on medicinal products for human use (HMD, Dir. 2001/83)^3^, regulation on veterinary medicinal products (VMR, Reg. 2019/6)^4^.

In the assessment of the environmental exposure and persistence of chemical and pharmaceutical substances, RBTs are a type of first-tier screening test. Originally introduced into regulatory testing more than 30 years ago, they have formed the basis of all biodegradability assessments^5^. This was largely due to their relatively low cost, perceived standardization, and straightforward implementation and interpretation of results. RBTs are considered stringent tests providing an assessment of the fate of chemicals in aerobic condition and aqueous media^5^.

The standards that can be used to perform RBTs are published by various international organizations such as AFNOR (Association Française de Normalisation), ISO (International Organization for Standardization), OECD (Organization for Economic Co-operation and Development), with those of the OECD being the most commonly used^6^. Currently, there are seven OECD RBTs identified by the numbers 301A-F^7^ and 310^8^.

They are pass/fail tests focusing on mineralization of the chemicals under analysis by measuring carbon dioxide evolution, oxygen consumption or removal of the dissolved organic carbon. The chemicals are tested at high concentrations (typically 10–100 mg/L) in a mineral medium, in the presence of a diluted microfauna (inoculum) collected from the environment. Biodegradation is monitored over 28 days^5^. The ready biodegradation carried out by the microfauna under standardized laboratory conditions should reproduce the fate and burden of anthropogenic organic chemicals in the environment, simulating the biodegradation that can transform potentially toxic chemicals into less as well as more harmful products^9^. During biodegradation, processes can be triggered which lead to the creation of more harmful products which can inhibit the microfauna itself and, therefore, interrupt biodegradation. The kinetic of this process can be strongly variable, depending on the ability of the microfauna to obtain the elements necessary for its survival.

RBTs are known to have a number of well-documented limitations, including the high number of failed tests and high variability that can negatively affect the chemical risk and hazard assessment, reported by Kowalczyk et al.^10^ Two main limitations can be highlighted: (1) it is not considered essential to monitor the RBT results with ad hoc analytic methods; it is assumed that if pass levels are fulfilled, the tested molecule is completely degraded into elementary building blocks; (2) the results obtained for a single pure compound are also considered valid in chemical mixtures, such as a pharmaceutical formulation. Recently it was demonstrated through untargeted and targeted analyses using UHPLC-qToF (an ultrahigh-performance liquid chromatograph coupled to a quadrupole time of flight mass spectrometer) that in pharmaceutical formulations, the mixture (i.e., the Active Pharmaceutical Ingredient, API, together with the excipients of the formulation) affects biodegradation of the API. Furthermore, it was also observed that the biodegradability of a chemical formulation can be a non-linear system and cannot be predicted as simply the sum of the biodegradability of each of its individual pure ingredients^11,12^.

Suspect screening and untargeted approaches based on high resolution mass spectrometry (HRMS) are now available for the chemical profiling and holistic characterization of complex mixtures, while targeted approaches are used to monitor a few selected chemical species. These advanced techniques, when applied to environmental analysis^13^, make it possible to detect a large number of chemical features simultaneously, and are currently being introduced into the study of contaminants of emerging concern (CECs)^14^ and the study of the extent of human chemical exposure (the exposome)^15^.

**Figure 1:**
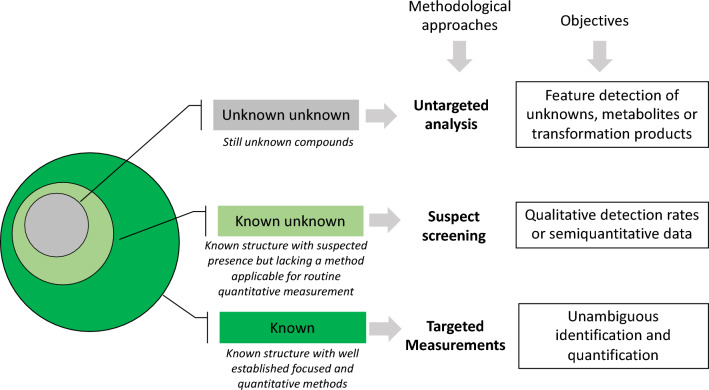
Conceptual view of compounds knowledge, methodology and objectives.

From a conceptual point of view, the three methodological approaches used to study the composition of complex samples from different sources can be defined as: (1) untargeted analysis, to track "unknown unknown" compounds; (2) suspect screening, to monitor “known unknown” compounds; and targeted measurements, to evaluate "known" compounds, or targets (3) (Fig. 1).

By definition, Untargeted analysis tries to detect “unknown unknown” compounds without definition of any a priori criteria. Compounds for which no identity can be readily assigned are termed “unknown unknowns”. These “un-targets” require further structural elucidation and can only be monitored by untargeted analyses. Therefore, untargeted approaches make it possible to detect many more compounds than can be identified.

”*Suspects*” are known compounds in terms of chemical name and structure which are expected to be present in a sample. The typical approach in this case is “suspect screening analysis”, which aims to generate semi-quantitative data for a wide range of compounds from each individual sample, helping to determine the composition of complex mixtures.

The annotation of “suspects” is a fundamental step. It refers to the assignment of an identity to a signal detected during the analysis and first relies on a match with a specific theorical or experimental database, reporting the list of compounds with structural data, such as their exact mass, the accurate mass of the compounds and their corresponding fragment ions, ion intensity, isotope spacing, etc. In suspect screening analysis, compounds for which no analytical standards are available are also considered. However, “s*uspects*” can potentially be later “converted” into “targets”, when the analytical standard becomes available and data can be collected which allows unequivocal identification of the *suspect* compound.

“Targets” are compounds of known chemical name and structure, for which analytical standards are available. Quantitative targeted methods can be validated and a number of guidelines to harmonize method performance assessment are available (e.g. Commission Decision 2002/657/EU, ICH Q2R2 validation of analytical procedures guideline, Eurachem guide, The Fitness for Purpose of Analytical Methods).

It is important to remember that these types of investigation can be conducted using high resolution mass spectrometers (e.g., UHPLC-qToF), which, operating in "all ions mode", simultaneously acquire data that can be used for multiple evaluations.

Against this background, and in keeping with our interest in the application of modern and advanced technologies for a more realistic and accurate evaluation of the biodegradability of chemical formulations (considering them as a complex systems)^11,12^, here a multifaceted analytical approach through untargeted, suspect screening and targeted methodologies using UHPLC-qToF is proposed. This comprehensive approach will help provide a deeper and more reliable understanding of the fate of each component in a complex mixture by determining the presence of metabolites produced during RBTs. Indeed, it is known that the determination of drug metabolites and transformation products is a problem of general interest for the determination of environmental contamination by drugs.

Although highly challenging, these approaches are the most promising strategy in advancing the understanding of complex mixtures, and thus the study of their biodegradation and environmental fate.

## Materials and methods

*Products tested* “Product A”, gastro-resistant capsules, the drug containing omeprazole, was purchased at a local pharmacy (batch n° LC52792); “Product B”, tablets, the natural substance-based medical device -Neobianacid- was produced by Aboca SpA (batch n° 21D1848); omeprazole pure standard was purchased from Sigma-Aldrich (from Merck KGaA, Darmstadt, Germany). The ingredients of “Product A” and “Product B” are listed below and are reported as they are in the leaflet.

Each gastro-resistant capsule of “Product A” contains 0.226 g of granules, with 20 mg of omeprazole. Other main ingredients are sugar spheres (consisting of corn starch and sucrose), sodium lauryl sulfate, anhydrous disodium phosphate, mannitol, hypromellose 6 cP, macrogol 6000, talc, polysorbate 80, titanium dioxide (E171) and copolymer of methacrylic acid- ethyl acrylate (1:1).

Each tablet of “Product B” -Neobianacid- weighs 1.55 g. “Product B” is a multicomponent natural complex product containing UVCB (Unknown or Variable Composition, complex reaction products or of Biological materials) substances of botanical and mineral origins, according to REACH^1^ and European chemical agency definitions^16^. “Product B” is defined by its peculiar, standardized manufacturing process and by its properties, verified according to an integrated analytical platform. The platform integrates NIR fingerprinting of the complex product with its physical and biophysical data, thus providing a product characterization which is then used to validate the production process. In terms of efficacy and safety, this platform guarantees reproducibility between different production batches (UIBM Patent application n. 102,023,000,005,949, filing date March 28th, 2023).

*Software* The structural formulas were designed using Biovia draw software (Dassault Systemes, version 20.1.NET, 2019), enterprise licence.

### Biodegradability test

#### Analytical approach of the test performed for “Product A” in a laboratory operating in accordance with GLP (Good Laboratory Practices). The activities were conducted following GLP principles.

Ammonium chloride, calcium chloride anhydrous, iron(III) chloride hexahydrate, magnesium sulphate heptahydrate, mercury chloride, potassium hydroxide, potassium phosphate dibasic anhydrous, potassium phosphate monobasic anhydrous, sodium phosphate dibasic dihydrate and sodium benzoate were purchased from Sigma Aldrich (from Merck KGaA, Darmstadt, Germany). Deionized water was prepared using the MilliQ water purification system (from Millipore, Merck KGaA, Darmstadt, Germany).

*Sample Preparation*: The sample was prepared by crushing it with a mortar and pestle. The test sample (0.0564 g) was added to 100 mL of medium. Next, the test item was used, with a final concentration corresponding to 56.4 mg/L, or 63.15 mg COD/L. Sodium benzoate was used as the reference substance. In every bottle so prepared, potassium hydroxide (a CO_2_ absorbent) was added to the CO_2_-absorber compartment. The bottles were then closed and incubation began, stirring under conditions of darkness, as described in the OECD 301F method. The culture medium with the addition of the inoculum was used as the blank. The test item was combined with 50 mg/L of HgCl_2_ in order to check any possible abiotic degradation.

*Inoculum preparation*. For the preparation of the inoculum, a sample of aerobic sludge was selected from the mixed treatment plant of urban (about 66%) and industrial (about 34%) liquid sewage. In the laboratory, the sludge samples were mixed and allowed to settle, keeping them in medium and aerobic conditions for 2 days. Before their use, the sludge samples had been centrifuged, washed and analysed in order to quantify their suspended solids concentration for preparation of the inoculum.

*Instrumental parameters*. The biological oxygen demand was constantly monitored by means of Oxitop Sensor System 6, made up of six Oxitop sensors, a six-position stirring base, dark glass bottles, sub-caps for the absorption of carbon dioxide and magnetic stir bars. Once a week, the values of all bottles on the automatic respirometer were recorded. All the experiments were conducted at a controlled temperature in a refrigerated thermostat equipped with an auto-tuning thermoregulation system and forced air circulation in order to ensure the stability and homogeneity of the internal temperature.

*Calculation.* The biodegradation was calculated at every sampling time for the test item bottle, subtracting the oxygen depletion of the blank sample. Total biodegradation was calculated using the following equations:

$$\% \;{\text{D}} = {\text{BOD }}\left( {{\text{mg}}\;{\text{O}}_{{2}} /{\text{mg}}\;{\text{test}}\;{\text{substance}}} \right)/{\text{COD }}\left( {{\text{mg}}\;{\text{O}}_{{2}} /{\text{mg}}\;{\text{test}}\;{\text{substance}}} \right) \times {1}00$$

where BOD: biochemical oxygen demand is the amount of oxygen consumed by microorganisms when metabolizing the test item; also expressed as mg oxygen uptake per Liter. These values are reported on the Oxitop system. COD: chemical oxygen demand is the total amount of oxygen required to oxidize a chemical completely. % D: biodegradation percentage of the test substance.

Values of ammonia, nitrates and nitrites were measured at the beginning and the end of the test and the results were considered in the BOD calculation.

#### Analytical approach of the test performed in a laboratory certified according to ISO 17,025

Magnesium sulphate heptahydrate for analysis ACS Reag. Ph.Eur and Reag. USP, sodium acetate anhydrous USP, phosphoric acid 99%, potassium phosphate dibasic 99 + % anhydrous for analysis, potassium phosphate monobasic 99% anhydrous for analysis, Iron(III) chloride hexahydrate Pure, sodium phosphate dibasic dihydrate 98 + %, calcium chloride anhydrous 99% pure, were purchased from Carlo Erba Reagents srl (*Cornaredo, Milano, Italy*). Ammonium chloride 99% puriss and potassium hydroxide 85% puriss were purchased from Italchimica SPA (*Pontecchio Polesine, Rovigo, Italy*). Purified water was prepared by Arium Mini water purification system (from Sartorius *Goettingen, Germany*).

*Sample Preparation*: The sample was prepared by crushing it with a mortar and pestle. In order to achieve the correct registration by the BOD sensor and have enough material to perform the instrumental analysis planned in a 1L vessel, 0.4 L of mineral medium and 50 mg/L or 75 mg/L of sample were introduced. Sodium acetate was used as reference substance. In every bottle so prepared, potassium hydroxide (a CO_2_ absorbent) was added to the CO_2_ absorber compartment. Next, the bottles were closed, and incubation began, stirring under conditions of darkness, as described in the OECD 301F method. Culture medium with the addition of the inoculum was used as the blank.

*Inoculum preparation*. The inoculum was prepared, collecting five samples of activated sludge and five samples of river water from different places and mixed in equivalent volume. The inoculum was oxygenated, stirred and fed with glucose, peptone and potassium phosphate dibasic. The values of redox potential, oxygen consumption and total dry matter were monitored daily. At the time of use, the dry substance was determined at 100 °C to measure the same quantity (30 mg/ml) in the vessels containing the test substance.

*Inoculum composition*. The inoculum was obtained by mixing active sludge from eight different zones and river water taken from two different rivers in equal parts. The assembled inoculum was oxygenated, stirred and fed with glucose, peptone and monopotassium orthophosphate. Oxygen, redox, and total suspended solids values were monitored daily.

Before the use of inoculum, the total dry matter was determined.

The composition of the microfauna was determined by optical microscopy and was composed of the following species: Rotifers 22.45%, Vorticella acquadulcis 20.41%, Aspidisca costata 14.29%, Vorticella convallaria 12.24%, Amoebas 12.24%, Tardigrades 10.20%, Litonotus Fasciola 8.16%.

*Instrumental parameters*. The biological oxygen demand was constantly monitored by BOD EVO Sensor System 6, made up of six BOD EVO sensors, a six-position stirring base, dark glass bottles, sub-caps for the absorption of carbon dioxide and magnetic stir bars. The BOD EVO sensor transmits the data wirelessly to the dedicated BODSoft ™ software (*VELP Scientifica, Usmate, Monza-Brianza, Italy*). All the experiments were conducted under controlled temperature in a refrigerated thermostat equipped with an auto-tuning thermoregulation system and forced air circulation, in order to ensure the stability and homogeneity of the internal temperature (*VELP Scientifica, Usmate, Monza-Brianza, Italy*).

*Calculations.* The biodegradation was calculated at every sampling time for the test item bottle, subtracting the oxygen depletion of the blank sample. Total biodegradation was calculated using the following equations:

$$\% \, = {\text{BOD}}\left( {{\text{mg}}\;\;{\text{O}}_{{2}} /{\text{mg}}\;\;{\text{test}}\;\;{\text{substance}}} \right)/{\text{COD }}\left( {{\text{mg}}\;\;{\text{O}}_{{2}} /{\text{mg}}\;\;{\text{test}}\;\;{\text{substance}}} \right) \, \times { 1}00$$

where BOD: biochemical oxygen demand is the amount of oxygen consumed by microorganisms when metabolizing the test item; also expressed as mg oxygen uptake per Liter. These values are reported on BODSoft ™ software. COD: chemical oxygen demand is the total amount of oxygen required to oxidize a chemical completely. % D: biodegradation percentage of the test substance.

Values of ammonia, nitrates and nitrites were measured at the beginning and end of the test, and the results were considered in the BOD calculation.

### UHPLC ESI-qToF methods

All solvents were of high purity analytical grade and were used without further purification. ULC/MS grade absolute methanol was purchased from Biosolve (Dieuze, France). Ultrahigh purified water was prepared in a PURELAB® Ultra water purification system (*ELGA, UK*). Formic acid 98–100% for LC–MS LiChropur® and dimethyl sulfoxide ≥ 99% were purchased from Sigma-Aldrich (from Merck KGaA, Darmstadt, Germany).

For the targeted study water LC/MS grade, methanol LC/MS grade, acetonitrile LC/MS grade and formic acid LC/MS grade were purchased from Sigma-Aldrich (from Merck KGaA, Darmstadt, Germany). Omeprazole and Omeprazole sulphide were purchased from Supleco (from Merck KGaA, Darmstadt, Germany).

#### Untargeted analytical methodology

*Sample preparation*: The sample was filtered on a 0.20 μm Millipore cellulose acetate syringe filter and was used to acquire the chromatographic profile without any dilution according to the conditions reported below. For “Product A” and “Product B” the test solution at 50 mg/L and 75 mg/L were used, as they assured the best compromise between sample dilution, signal quality and peak abundance of the various compounds.

##### Multivariate statistical analysis

*Data collection*. Samples of the product at T0 and T28 and the blank sample, consisting of mineral medium and inoculum, were organized into a randomized sample queue prior to UHPLC-qToF acquisition. The analytical blank and pooled samples were acquired at the beginning, middle and end of the sample sequence. Each sample was acquired in triplicate. For “Product “A instrumental parameters were those used for the suspect screening (positive ions), while for for “Product B” are reported hereinafter.

The analytical column was a Cortecs® C18 (100 × 2.1 mm, 1.6 μm) protected by a Cortecs® UPLC® C18 VanGuard™ pre-column (5 × 2.1 mm, 1.6 μm) both supplied by Waters (Milford, MA) and the thermostat was set at 40 °C. The analyses were performed in elution gradient (Table SI-1) using aqueous HCO_2_H (0.1%, v/v) as mobile phase A and HCO_2_H (0.1%, v/v in MeOH) as mobile phase B, with a flow rate of 0.3 mL min^-1^. The volume injected was 10 μL. The UHPLC system was coupled to a q-ToF mass spectrometer equipped with a Dual AJS ESI source operating in negative ionization mode, with a scan range from 50 to 1700 m*/z*. The optimized instrument parameters are reported in TableSI-2.

*Data processing and data analysis*. Peak peaking and peak alignment were obtained using the software R [R version 3.5.2 (2018-12-20), Copyright (C) 2018 The R Foundation for Statistical Computing]. Mass tolerance was set at 10 ppm and retention time tolerance was set at 10 s. Signal Filtering Threshold was set at 1000 counts. Variables with a frequency of missing values and coefficient of variation greater than 20% were eliminated and data imputation was performed. Additionally, a filter to eliminate those variables present in the analytical blank samples was applied.

Median of the replicas was applied to remove the effect of the random noise, while data normalization was performed by centering and scaling with PQN (Probabilistic Quotient Normalization) on reference pooled samples.

The processed data were saved in CSV format and the data matrix in CSV format has been reworked by the program SIMCA (Version 16.0.2.10561, Jan 22, 2020, Umetrics/Sartorius). Later, the data matrix was mean centered and Pareto scaling was applied prior to performing the data analysis.

Statistical Model performance evaluation was carried out and a confidence level of 95% was used. Goodness of fit and goodness of prediction were evaluated by means of Q^2^X and R^2^X, respectively. The models obtained from the data of “Product A”, at a concentration of 50 mg/L, showed a Q^2^X value of 0.948 and R^2^X of 0.985, while for “Product B”, at a concentration of 75 mg/L, Q^2^X was 0.948 and R^2^X was 0.985. The diagnostic tools Hotelling T^2^ and dModX were calculated to be sure that the model was not deformed. The models were robust, as the observations were below the calculated critical values and there were no outliers (Fig. SI-1).

*PCA analysis*. After data processing, in order to evaluate the complex fingerprints obtained, multivariate analysis of the Principal Component (PCA) was used. The PCA model was built using the first two principal components, providing a 2D model (Confidence level 95%).

*Cluster analysis.* Cluster analysis was performed according to an unsupervised approach using a hierarchical algorithm (Confidence level 95%). Distance indices were determined using Ward's method.

*OPLS-DA analysis*. After data processing, in order to evaluate the behaviour of the three classis (Class 1 = mineral medium; Class 2 = Product A at T0; Class 3 = Product A at T28), multivariate analysis of the orthogonal partial least square-discriminant analysis (OPLS-DA) was used. The OPLS-DA model was built using the autofit to maximise the predictivity of the model (Q^2^). Statistical model performance was evaluated by means of permutation test. A three-fold cross validation was imposed. The corresponding model was characterized by two predictive components (CV_*Anova*_, *p*-value = 2.15^–10^).

#### Suspect screening analytical methodology

*Sample preparation*: Samples were used to acquire the chromatographic profile, after filtration on a 0.20 μm Millipore cellulose acetate syringe filter. The samples containing the test item were injected as it is and after dilution 1:10 and 1:50 in mineral medium, to evaluate the ionization yield and permit the subsequent semi-quantitative determination. Blank, samples and standard have been also filtrated and 1 mL of each solution has been placed in vial with internal standard.

The “Product A” test solution at 50 mg/L was analyzed, as it assured the best compromise between sample dilutions, signal quality and peak abundance of the various compounds.

*Instrumental parameters*. The instrumental platform used consisted of an UHPLC series 1290 coupled to a quadrupole time-of-flight (q-ToF) mass spectrometer series 6545 (Agilent Technologies, Santa Clara, CA) in high resolution (2 GHz). The UHPLC was equipped with a binary pump, an autosampler, a multicolumn thermostat, an isocratic pump and a solvent cabinet. The autosampler was maintained at 15 °C.

For “Product A”, the analytical column was a ACQUITY UPLC BEH® C18 (100 × 2.1 mm, 1.7 μm) protected by a ACQUITY UPLC BEH UPLC® C18 VanGuard™ pre-column (5 × 2.1 mm, 1.6 μm) both supplied by Waters (Milford, MA) and the thermostat set at 40 °C.

The analyses were performed in elution gradient (Table SI-1) using aqueous HCO_2_H (0.1%, v/v) as mobile phase A and HCO_2_H (0.1%, v/v in MeOH) as mobile phase B, with a flow rate of 0.3 mL min^−1^. The volume injected was 10 μL.

The UHPLC system was coupled to a q-ToF mass spectrometer equipped with a Dual AJS ESI source operating in positive ionization mode for “Product A” with a scan range from 50 to 1700 m*/z*. The optimized instrument parameters are reported in Table SI-2.

The acquisition of the sample fingerprint under investigation was performed in “All-Ions mode”^17,18^ using 30 eV of collision energy value. A reference mass solution containing purine and hexakis (1H, 1H, 3H-tetrafluoropropoxy) phosphazine was injected directly into the ESI source by the isocratic pump and ionized together with the sample solution for mass correction, making it possible to obtain accurate mass time-of-flight data.

*Software*. MassHunter software version B.07 (*Agilent Technologies, Santa Clara, CA*) was used for data acquisition and processing. The exact mass of the various molecular species was calculated by means of *Isotope distribution calculator* a tool with a MassHunter Data Analysis core, version 8.0.8208.0 (*Agilent Technologies, Santa Clara, CA*). The Metlin library used was Metlin_Metabolites_AM_PCDL, version 7.0.

*Data Processing and Data Analysis*. Computations were performed using the “Qualitative analysis” program and the “Find-by-Formula” algorithm. The data file is loaded into the “Qualitative analysis” then “Find-by-Formula” is run on the low channel against an MS/MS library. Find-by-Formula returns possible precursor formulas found in the library, as well as their product ions. Using the list of product ions, “Qualitative analysis” extracts EICs from the high channel and aligns them with an EIC of the precursor. A coelution score is calculated and compounds which pass the threshold (user-set at 70) are retained.

#### Targeted analytical methodology

##### Parameters of the test performed for “Product A” in a laboratory operating in GLP. The activities were conducted according GLP principles.

*Sample preparation*: Samples were used to acquire the chromatographic profile, after filtration on a 0.20 μm Millipore cellulose acetate syringe filter. The samples containing the test item were injected as it is and after dilution 1:10 and 1:50 in mineral medium, to permit the correct quantitative determination. Blank, samples and standard have been also filtrated and 1 mL of each solution has been placed in vial with internal standard.

*Data collection*. Samples of the “Product A” -concentration 50 mg/L- at t0 and T28 and the blank sample, consisting of mineral medium and inoculum, were analysed by UHPLC-qToF. Each sample was acquired in triplicate. Instrumental parameters were those used for the suspect screening of “Product A”.

*Method Validation.* The aim was to validate the analytical method to quantify omeprazole (CAS number 73590-58-6) and omeprazole sulphide (CAS number 73590-85-9) in OECD 301F Medium. The parameters validated were specificity, accuracy at three level, precision, linearity, LoQ (limit of quantitation).

Specificity was evaluated, demonstrating that the blank, and the matrix solutions does not produce any peak interfering with the analytes. Range was assessed by accuracy verification. The linearity of the method was assessed by analysing at least five standard solutions over the range of validation. The analyte concentration was plotted against its instrumental response. For the resulting interpolation line, the correlation factor, slope and intercept were calculated, and the acceptance criterion for correlation was R^2^ > 0.99. Method precision was checked calculating the % of RSD (relative standard deviation) of recoveries obtained at each of the three levels considered (high level, medium level, low level), on the spiked samples prepared for accuracy evaluation. Method accuracy was calculated as recovery percentage of the assay of three spiked samples. The recovery should be between 80 and 120% with relative standard deviation (RSD) ≤ 20%. The limit of quantification (LoQ) value is usually assessed on standard solution chromatograms as the concentrations giving an S/N ratio of approximately 10. Since this value was lower than the concentration established in the linearity test for both omeprazole and omeprazole sulphide (TP-9), the last concentration of the linearity test was considered as the LoQ.

## Results and discussion

In order to apply our theories and demonstrate the efficiency of the here proposed analytical approach to real samples, the investigation was performed using two formulations available and largely distributed on the market for the treatment of reflux diseases and functional dyspepsia as “proton pump inhibitor (PPI)” (Product A) or “oesophageal and gastric mucosal protectors” (Product B). “Product A” contains synthetic non-naturally occurring ingredients and “Product B” contains only natural ingredients. “Product A” is based on 5-Methoxy-2-[(4-methoxy-3,5-dimethylpyridin-2-yl)methanesulfinyl]-1*H*-benz[*d*]imidazole (omeprazole®); “Product B” (neobianacid®) is a medical device certified according to the European legislation. It is a complex multiple component product containing natural matrices resulting from a standardized manufacturing process. Its biological activity relies on its physicochemical properties, which are also clearly established by the weak bonds between the constituents in its supramolecular organisation^19^.

Very recently the clinical efficacy and safety of “Product B” compared to “Product A” were evaluated in a double-blind, double-dummy, randomized non-inferiority clinical trial^20^. A more accurate method for the evaluation of the biodegradability of complex mixtures should contribute to better profiling the fate of the ingredients composing a pharmaceutical formulation enabling to determinate a more realistic environmental impact giving parameters for a more accurate determination of the risk–benefit ratio of equivalent therapeutic solutions.

The manometric respirometry test OECD 301F^8^ was chosen as the method for the ready biodegradability evaluation of the two formulations, as one of the methods most frequently used to test complex mixtures^21^. This method was also reported as method C.4-D by regulation 440/2008^22^, which established the test methods according to regulation 2006/1907 (REACH)^1^. “Product A” and “Product B” were placed in a mineral medium in the presence of the inoculum. The inoculum used was obtained from samples of activated sludge and surface water, mixed in equal parts and characterized in its macro constituents. It wasn’t preadapted to the tested mixtures, so the results can be considered representative and robust. The oxygen consumption was recorded using a calibrated continuous reading probe. Three replicas were performed for each product, to ensure the repeatability of the results. At the concentration of 50 mg/L, "Product A" was not readily biodegradable, both because it does not exceed the 10-day window criterion and because 60% biodegradation is not reached on the 28th day. The test, repeated according to GLP principles at a concentration of 56.4 mg/L, confirmed that it was not readily biodegradable, as the 10-day criterion was not satisfied, and biodegradation reached 33% at the 28th day (Table 1). The differences observed are to be attributed to the biological variability of the test. “Product B”, tested at a concentration of 50 mg/L, resulted readily biodegradable, as they passed the 10-day window criterion and reached 91% of biodegradation on the 28th day, a result which was also confirmed at 75 mg/L.

**Table 1:** Results of the OECD 301F ready biodegradability test.

Product	Concentration (mg/L)	Ten-day window criterion	Biodegradation on the 28th day^1^	Result
Omeprazole, pure standard	4.4	Not passed	2%^2^	Not readily biodegradable
Product A	56.4	Not passed	33%^3^	Not readily biodegradable
	50	Not passed	26%^2^	Not readily biodegradable
Product B	50	Passed	91%^2^	Readily biodegradable

^1^The pass level for ready biodegradability test with the manometric respirometry method OECD 301F is the achievement of 60% of theoretical oxygen demand (ThOD) reduction. This pass value must be reached in a 10-day window within the 28-day period of the test. The 10-day window begins when the degree of biodegradation has reached 10% of the ThOD. Chemicals which do not reach both pass levels are not considered readily biodegradable.

^2^Result of the test performed in an ISO 17,025 certified laboratory, where all the activities were managed according to ISO17025 principles.

^3^Result of the test performed in a GLP test laboratory, where all the activities were managed according to GLP principles.

For both “Product A” and “Product B”, the RBT result is the consequence of the ability of the microfauna to use the various organic compounds of the mixture as a source of carbon for its survival. In “Product A” there is Omeprazole, a synthetic organic compound reported to not be readily biodegradable. As described in the environmental risk assessment documents held at IVL Swedish environmental research institute^23^ and at the food and drug administration (FDA)^24^, after 28 days under the condition of the OECD 301C test, omeprazole biodegradation was lower than 0.6%. Similar results for pure omeprazole were obtained which, at the concentration of 4.4 mg/L (Table 1), resulted 2% biodegraded, in our case chosen to be directly comparable with that of omeprazole in “Product A” during the RBT.

A check of the European chemicals agency (ECHA) database showed that the environmental fate of omeprazole is not reported, while there are incomplete data for esomeprazole, the *S*-enantiomer of omeprazole^25^.

### Omeprazole human metabolites

Despite the extensive consumption, omeprazole is seldom detected in water, as it is almost completely metabolized by the human cytochrome P450 system. Some of the main metabolites reported are hydroxy-omeprazole, omeprazole sulphide and omeprazole sulphone derivatives^26,27^. It has also been shown that omeprazole metabolites and a series of their derivatives may enter and are likely to persist in aquatic environments^28^. Hence, to fully assess the environmental exposures and risks associated with omeprazole, it is important to better understand and evaluate the fate and behavior not only of the original compound but also its metabolites and transformation products (TPs) arising from abiotic processes in the environment^28^. A similar situation can be described for other members of the PPI drug class such as esomeprazole, lansoprazole, pantoprazole and rabeprazole, which are not regularly detected in environmental monitoring studies^28^.

### UHPLC-qToF analysis

Taking all these aspects into account, it is evident that applying advanced analytical technologies and methodologies to study the mixtures obtained at the end of the ready biodegradation tests in depth can provide useful information on the fate of each single compound that constitutes a complex mixture, such as a medicine or medical device. Therefore, the mixture obtained on the 28th day of RBT for “Product A” and “Product B”, under OECD 301F test conditions, was investigated by UHPLC-qToF analysis following untargeted, suspect screening and targeted approaches. In order to preserve the integrity of the sample and reduce the effect of the sample preparation, the mixtures were only opportunely diluted, filtered and injected into UHPLC-qToF.

The UHPLC-qToF method, used to acquire data, was characterized by a chromatographic separation in a reverse phase UHPLC C18 column followed by qToF mass spectrometer detection. The ESI source was used to produce the various ion molecular species, choosing the best ionization mode according to the ionization yield of the compounds present. The positive ion mode was used to analyze “Product A”, due to the presence of the nitrogenous basic compound Omeprazole. While the ionization in negative ion mode was applied in the case of “Product B”, as it was rich in phenolic compounds. The mobile phase was composed of water/methanol, with a gradient composition from a high percentage of water to a high percentage of methanol, thus satisfying the separation of most of the analytes and limiting the matrix effects. A final wash of the column with 99% methanol was introduced, to avoid carry-over between injections. Formic acid, as phase modifier, was added to the mobile phase to stabilize the pH, increase peak shape and promote ionization. To ensure repeatability and reach a better confidence level in the results produced, each sample was acquired in triplicate and pooled samples were acquired at the beginning, middle and end of the analytical session.

Finally, post-acquisition data processing allowed the raw instrumental data to be transformed into clean and normalized data, which were subsequently used for the multivariate statistical analysis and annotation phases. Targeted analysis was performed after validation of the chromatographic method to obtain robust and reliable quantitative data for omeprazole and omeprazole sulphide (TP-9).

### Untargeted analysis

**Figure 2:**
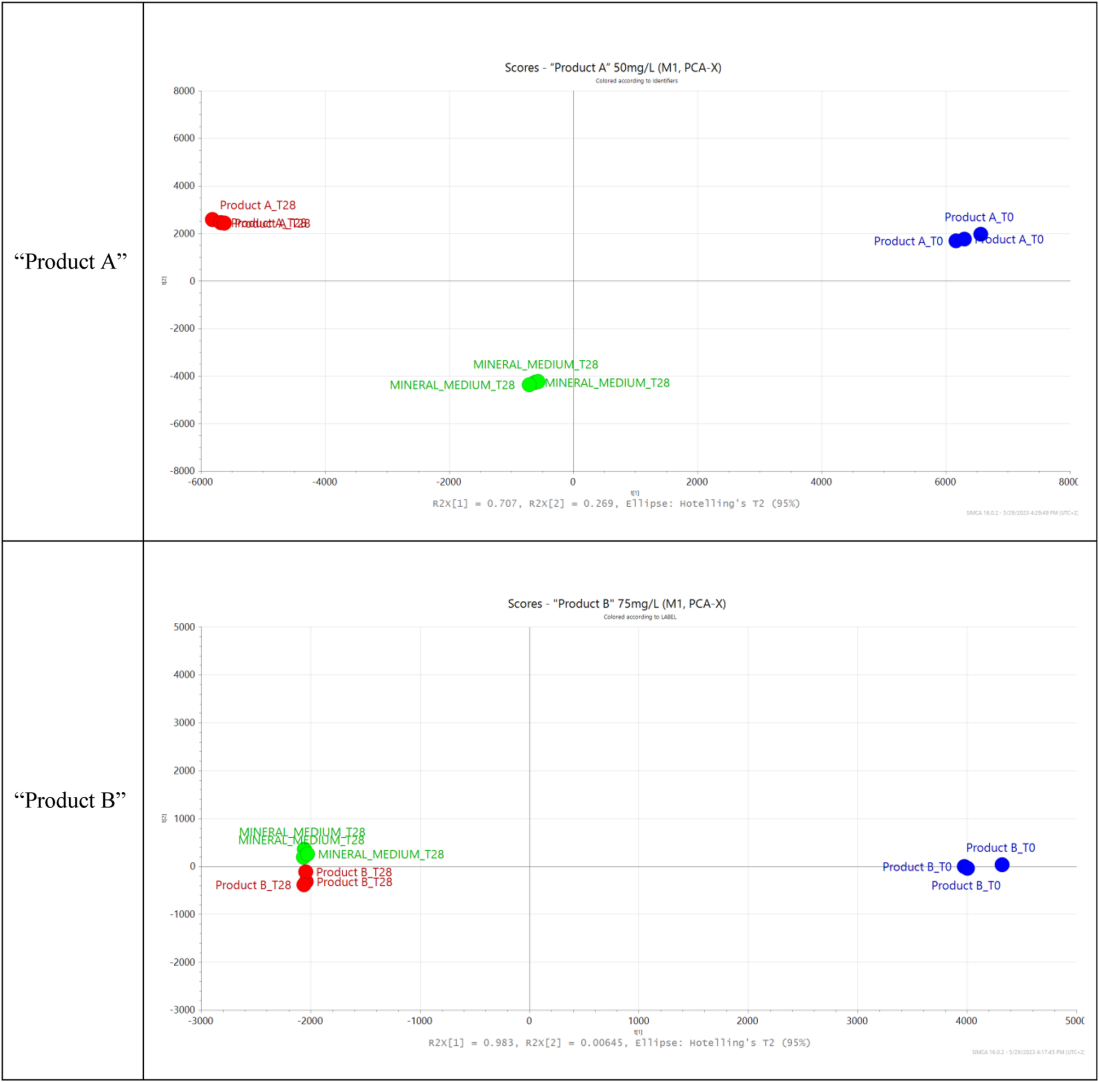
PCA models for “Product A” and “Product B”, from the ready biodegradability test OECD 301F.

Untargeted analysis was performed to obtain a holistic overview of the chemical fingerprint of the mixtures obtained from the RBT, visualizing the results after unsupervised multivariate statistical analysis. The analytical protocol used to record fingerprints and to perform data mining followed the suggestions provided by the metabolomic standard initiative (MSI) for performing metabolomic studies on complex matrices^29^. In practice, peak picking, peak alignment and peak integration were performed, respectively, to align common peaks found in the different samples and report their intensity or area. Variables with a frequency of missing values and a coefficient of variation greater than 20% were eliminated; noise was subtracted; the corresponding raw data matrix was subjected to normalization and scaling. The resulting peak list is characterized by a series of features, labeled with retention time and high-resolution mass. The resulting data matrix was used to build the statistical model, after posing the classical tests R^2^X, Q^2^ (model diagnostic tests), Hotelling T^2^ and DModX (observation diagnostic tests) at a 95% confidence level. The covariance matrix and standardized principal components were selected for unsupervised principal component analysis (PCA) computation. As can be seen in the 2D diagrams (Fig. 2), the first two components of the 2D-PCA were found to comprise more than 95% for “Product A” (PC1 70.7%, PC2 26.9%) and for “Product B” (PC1 98.3%, PC2 0.6%) of the total variance of the model.

The graphics evidenced that for “Product A” the observations (or score) are far from that of the mineral medium at 28th day while in the case of “Product B” the biodegradation appears to be quite fully coincident with mineral medium at the end of the test (T28). These results are very interesting and somehow confirm the different behavior of the two products in the biodegradation test, as shown in Table 1.

Another classical unsupervised investigation such as hierarchical cluster analysis (HCA) was performed. Because samples cluster in the same way as PCA, HCA plots are not reported. Data mining can be performed also according to untargeted supervised approach such as: PLS, PLS-DA, OPLS, OPLS-DA^30^. Among these, OPLS-DA was chosen because it can be very useful for obtaining significant information, especially for establishing which TPs have the greatest importance in the clustering of the samples. After verifying the predictivity of the model using the permutation test (Figure SI-2) it was seen that the OPLS-DA model did not add any new information compared to that of the PCA. However, through the loading plot (Figure SI-2) important loadings were highlighted in the clustering of the samples present in the model, some of which were identified and studied in the subsequent suspect screening analysis.

### Suspect screening analysis

Given the low percentage of biodegradation and the results of the non-targeted investigation, it was decided to investigate the biodegradation of “Product A” with a suspect screening approach. This study was not considered necessary for “Product B” because it is 100% natural and, due to its physicochemical characteristics, it is unlikely to represent a risk for the environment. In fact, even the legislation on medicines recalls that when an API is present in nature it is unlikely that it will pose a risk to the environment^3,31,32^.

In suspect screening analysis, the annotation of the suspect compound is a crucial step. It should be noted that the elucidation of small molecules using HRMS-based non-target and suspect analysis is relevant in various fields, for example in metabolomics^33^ and in environmental sciences^34^. Therefore, depending on the type and extent of structural information collected and available through the developed analytical workflow, compounds can be identified at various levels of confidence. Identified structures confirmed by reference standards are at level 1, then probable structures identified by library and literature spectrum data match and diagnostic evidence are at level 2. Further three levels describe grey zones for which structural identification is increasingly uncertain. At level 3 there are tentative candidates for which evidence exists for possible structure(s), but insufficient information for one exact structure only (e.g., positional isomers) is present. At level 4 unequivocal molecular formula can be unambiguously assigned while at level 5 only the exact mass of interest can be considered^33,34^.

In our case, the raw data collected at the beginning of the RBT and after 28 days were background subtracted and matched with the data found in the Metlin (Metabolites_AM), a commercially available database containing accurate mass and accurate MS/MS fragments for a wide range of pharmaceutical compounds and their metabolites. The library is integrated in the “qualitative analysis” of Mass Hunter (Agilent) and by applying the “find by formula” mining algorithm, it was matched with RBT data from “Product A”.

**Figure 3:**
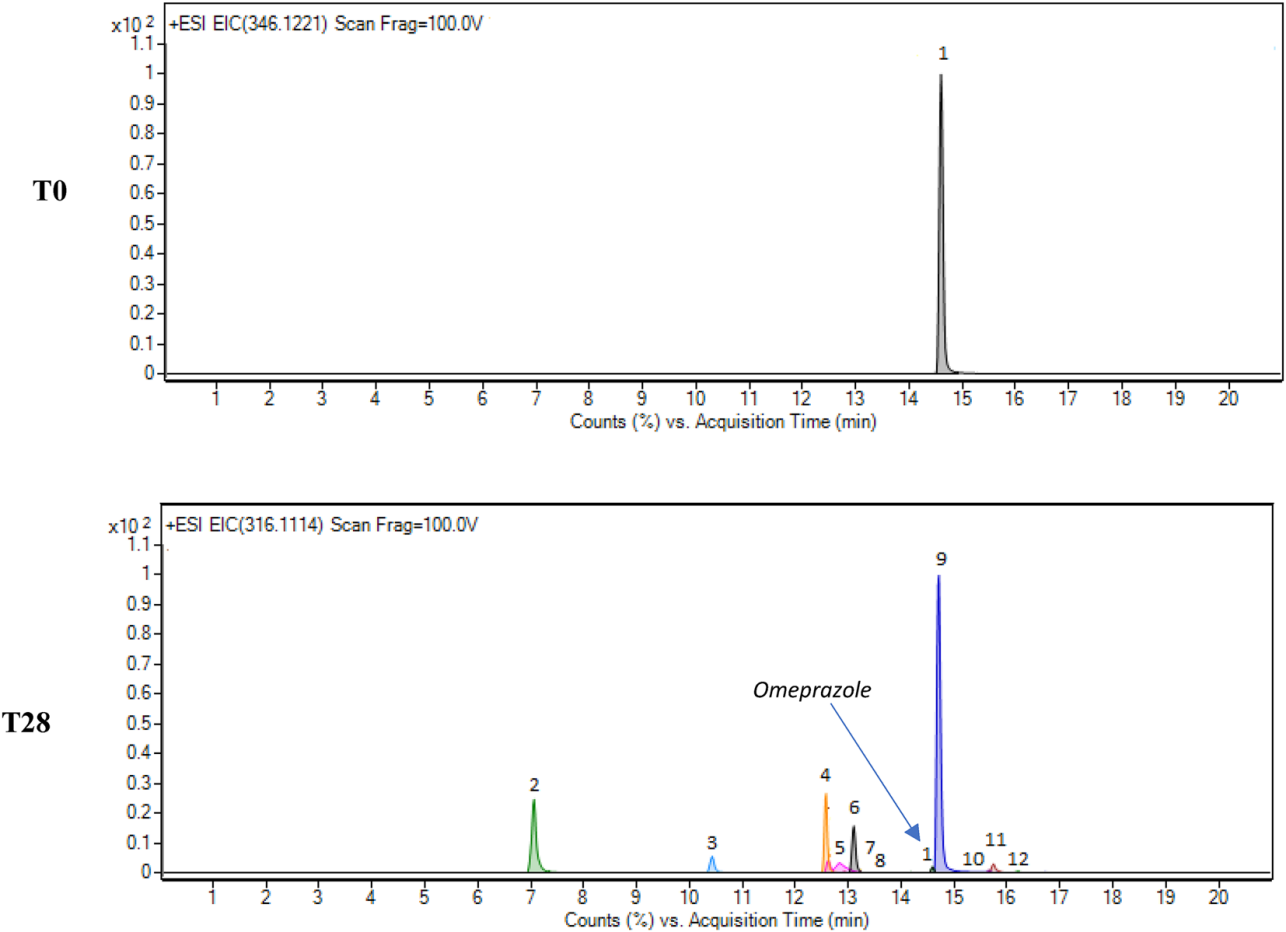
Biodegradation gradient showed by “Product A”. EICs at T0 and T28 for omeprazole (1) and its degradation products (TPs 2-12), from the RBT OECD 301F.

At the beginning of the RBT (T0), in the “Product A” mixture, omeprazole was detected (Fig. 3), in agreement with its the composition, then high-resolution mass, retention time, high resolution MS/MS fragmentation patterns, isotopic spacing and isotopic abundance of omeprazole were in agreement with data from a certified reference standard (Table 2).

**Table 2:** Omeprazole transformation products (TPs) formed during ready biodegradability test OECD 301F.

Compound_number IUPAC name	Molecular structure	Identification confidence^34^	Retention time, min	Elemental composition of ions	Exact calculated Mass of ions (m/z)	Accurate Mass of ions (m/z)	Mass error, ppm	Information about omeprazole TPs [Ref.]
**Omeprazole (1)** 6-methoxy-2-[(4-methoxy-3,5-dimethylpyridin-2-yl)methylsulfinyl]-1H-benzo[d]imidazole		1	14.601	[C_17_H_20_O_3_N_3_S]^+^ [C_9_H_12_O_2_NS]^+^ [C_9_H_13_ON]^+^ [C_8_H_9_ON_2_]^+^ [C_8_H_10_ON]^+^	**346.1221** 198.0583 151.0991 149.0709 136.0757	**346.1230** 198.0591 *151.0995 149.0713 136.0762	2.6 4.0 2.6 2.7 3.7	
**Omeprazole TP-2** 4-methoxy-3,5-dimethyl-pyridine-2-carboxylic acid		2	7.062	[C_9_H_12_O_3_N]^+^ [C_8_H_12_O_2_N]^+^ [C_8_H_10_ON]^+^	**182.0812** 154.0863 136.0757	**182.0816** 154.0865 136.0760*	2.2 1.3 2.2	Proposed secondary transformation product by photolysis in water^36^
**Omeprazole TP-3** 6-Methoxy-1*H*-benzo[*d*]imidazole-2-ol		2	10.430	[C_8_H_9_O_2_N_2_]^+^ [C_7_H_6_O_2_N_2_]^+^	**165.0658** 150.0423	**165.0661** 150.0425*	1.8 1.3	Proposed primary transformation product by hydrolysis in water, under neutral conditions^37^
**Omeprazole TP-4** 6-Methoxy-2-(4-methoxy-3,5-dimethyl-2-methylene-1-pyridyl)-1*H*-benzo[*d*]imidazole		2	12.579	[C_17_H_20_O_2_N_3_]^+^ [C_16_H_17_O_2_N_3_]^+^ [C_15_H_14_O_2_N_3_]^+^ [C_9_H_12_ON]^+^ [C_8_H_7_ON_2_]^+^ [C_8_H_10_N]^+^	**298.1560** 283.1315 268.1081 150.0913 147.0552 120.0808	**298.1555** 283.1315 *268.1081 *150.0914 147.0556 120.0810*	-1.7 0.0 0.0 0.7 2.7 1.7	Proposed primary transformation product by hydrolysis in water, under acidic conditions^37^
**Omeprazole TP-5** 1-(6-methoxy-1*H*-benzo[*d*]-imidazole-2-yl)-5-methyl-3-methylene-4-oxo-2*H*-pyridine-2-carboxilic acid		2	12.630	[C_16_H_16_O_4_N_3_]^+^ [C_16_H_14_O_3_N_3_]^+^ [C_15_H_16_O_2_N_3_]^+^ [C_15_H_14_O_2_N_3_]^+^ [C_14_H_13_O_2_N_3_]^+^ [C_7_H_8_ON]^+^	**314.1135** 296.1029 270.1237 268.1080 255.1002 122.0600	**314.1139** 296.1030 270.1238 268.1084 *255.1006 122.0603*	1.3 0.3 0.4 1.5 1.6 2.5	Proposed primary transformation product by hydrolysis in water, under neutral conditions^37^
**Omeprazole TP-6** 6-methoxy-2-[(3,5-dimethylpyridin-2-yl)methylsulfinyl]-1*H*-benzo[*d*]imidazole		2	13.104	[C_16_H_18_O_2_N_3_S]^+^ [C_15_H_15_O_2_N_3_S]^+^ C_15_H_14_ON_3_S]^+^ [C_8_H_10_ONS]^+^ [C_8_H_9_ON_2_]^+^ [C_8_H_10_ON]^+^	**316.1114** 301.0879 284.0852 168.0477 149.0709 136.0757	**316.1128** 301.0878 284.0853 168.0483 *149.0713 *136.0762*	4.4 -0.3 0.3 3.6 2.7 3.7	Proposed primary transformation product by hydrolysis in water, under basic and neutral conditions^37^
**Omeprazole TP-7** Pyridine ring hydroxylated and oxidated derivative of 6-methoxy-2-[(4-methoxy-3,5-dimethyl-pyridin-2-yl)methylthio]-1*H*-benzo[*d*]imidazole		3	13.239	[C_17_H_18_N_3_O_4_S]^+^ [C_17_H_17_N_3_O_4_]^+^ [C_9_H_10_NO_3_S]^+^ [C_9_H_10_NO_3_]^+^	**360.1018** 327.1219 212.0381 180.0661	**360.1015** 327.1205 *212.0380 *180.0664	-0.8 -4.3 -0.5 1.7	Proposed omeprazole urine metabolite, already found in wastewater and surface water^36,38^
**Omeprazole TP-8** Pyridine ring hydroxylated derivative of 6-methoxy-2-[(4-methoxy-3,5-dimethyl-pyridin-2-yl)methylthio]-1*H*-benzo[*d*]imidazole		3	13.290	[C_17_H_20_N_3_O_3_S]^+^ [C_17_H_19_N_3_O_3_]^+^ [C_9_H_12_NO_2_S]^+^ [C_8_H_10_NO_2_]^+^ [C_8_H_12_NO]^+^	**346.1225** 313.1426 198.0589 152.0712 138.0919	**346.1219** 313.1414 198.0588 *152.0708 *138.0918	-1.7 -3.8 -0.5 -2.6 -0.7	Proposed omeprazole urine metabolite, already found in wastewater and surface water. It was proposed that it could be partially glycosylated^38^
**Omeprazole TP-9** 6-methoxy-2-[(4-methoxy-3,5-dimethylpyridin-2-yl)methylthio]-1*H*-benzo[*d*]imidazole (ufiprazole)		1	14.711	[C_17_H_20_N_3_O_2_S]^+^ [C_17_H_19_N_3_O_2_]^+^ [C_16_H_17_N_3_O_1_]^+^ [C_9_H_12_NOS]^+^ [C_8_H_8_NOS]^+^ [C_9_H_12_NO]^+^ [C_8_H_9_N_2_O]^+^ [C_8_H_10_NO]^+^ [C_8_H_10_N]^+^	**330.1270** 297.1471 267.1366 182.0634 166.0321 150.0913 149.0709 136.0757 120.0807	**330.1283** 297.1477 *267.1365 182.0639 *166.0319 150.0920 149.0717 *136.0762 120.0815	3.9 2.0 -0.4 2.7 -1.2 4.6 5.3 3.7 6.6	Proposed primary transformation product by hydrolysis in water, under acidic and basic conditions^37^ This compound was also described as mouse brain and plasma metabolite of omeprazole^40^
**Omeprazole TP-10** Pyridine ring hydroxylated derivative of 6-methoxy-2-[(4-methoxy-3,5-dimethylpyridin-2-yl)methylsulfinyl]-1*H*-benzo[*d*]imidazole		3	15.659	[C_17_H_20_N_3_O_4_S]^+^ [C_8_H_7_N_2_O_2_S]^+^ [C_9_H_12_NO_2_]^+^ [C_9_H_12_NO]^+^ [C_8_H_9_N_2_O]^+^ [C_8_H_10_N]^+^	**362.1169** 195.0222 166.0868 150.0913 149.0709 120.0807	**362.1171** 195.0218 *166.0872 *150.0917 149.0718 120.0811	0.6 -2.0 2.4 2.0 6.0 3.3	Proposed primary transformation product by oxidative degradation in water^37^ This compound was also described as mouse brain and plasma metabolite^41^ and as human urine metabolite found in wastewater^36,38^
**Omeprazole TP-11** 6-methoxy-2-[(3,5-dimethylpyridin-2-yl) methoxy]-1*H*-benzo[*d*]imidazole		2	15.744	[C_16_H_18_N_3_O_2_]^+^ [C_15_H_15_N_3_O_2_]^+^ [C_15_H_14_N_3_O_2_]^+^ [C_14_H_12_N_3_O_2_]^+^ [C_8_H_7_N_2_O]^+^ [C_8_H_10_NO]^+^	**284.1393** 269.1158 268.1080 254.0924 147.0552 136.0757	**284.1397** 269.1163 *268.1076 254.0930 *147.0555 136.0761	1.4 1.8 -1.5 2.4 2.0 2.9	Proposed primary transformation product by hydrolysis in water, under neutral conditions^37^
**Omeprazole TP-12** 4,13-dimethoxy-12,14-dimethyl-1,8,10-triazatetracyclo[7.7.0.0^2,7^.0^10,15^]hexadeca-2(7),3,5,8,11,13-hexaen-16-one		2	16.218	[C_17_H_18_N_3_O_3_]^+^ [C_16_H_15_N_3_O_3_]^+^ [C_15_H_15_N_3_O_2_]^+^ [C_15_H_14_N_3_O] ^+^	**312.1342** 297.1107 269.1158 252.1131	**312.1352** 297.1107 *269.1161 252.1126	3.2 3.0 1.5 -2.0	Proposed primary transformation product by hydrolysis in water, under acidic and basic conditions^37^

In bold are reported the molecular ions. The font * indicate the more abundant ions.

Mass error in part per million was calculated by means of mass calculator^35^.

The same search was carried out on the sample on the 28th day (T28). From the analysis of these data, it emerged that omeprazole (peak 1) was still detected, as evidenced by the extract ion chromatograms (EIC) of the precursor ions in Fig. 3, in agreement with its well-known low biodegradability^23,24^.

At the same time, the results of the research carried out on Metlin have highlighted the presence of 5-Hydroxy-omeprazole, which can be one of the possible structures of our TP-10 (Table 2). The lack of information led us to manually search for Omeprazole TPs already described in the literature. The results of this search made it possible to tentatively identify eleven different compounds shown in Table 2, whose chromatographic peaks are represented in Fig. 3. Among all the tentative TPs, only for omeprazole sulphide (ufiprazole, TP-9) was confirmed the identification by acquiring the chromatographic and spectral data of the certified reference standard with the same analysis methods.

A study was carried out to check for the presence of common ions among the metabolites formed during RBT. Each compound reported in Table 2 presents a typical fragmentation pattern, however the fragment ion at m/z 136.0757 is common to compounds **2**, **6**, **9**, **11** and to omeprazole itself. The tentative elemental composition of this fragment is [C_8_H_10_NO]^+^ which is related to the 4-methoxy-3,5-dimethylpyridinyl moiety of the molecular structure of omeprazole.

**Figure 4:**
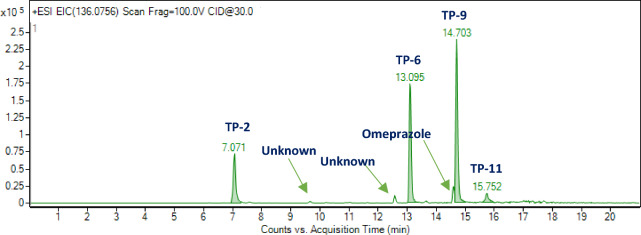
Extract Ion Chromatogram of the ion at m/z 136.0757.

The extract ion chromatogram of another common ion at m/z 136.0757 (Fig. 4) revealed not only the presence of compounds **2**, **6**, **9** and **11**, but also the presence of other small chromatographic peaks, emphasizing that the mixture is more complex and that other unidentified TPs of omeprazole are also present.

Another interesting fragment ion is that at m/z 149.0709, which has the elemental composition [C_8_H_9_ON_2_]^+^. It is common to compounds **6**, **9** and **10** and can be related to 5-methoxy-benzoimidazolyl moiety.

Unfortunately, the 5-methoxy-benzoimidazolyl moiety ionizes differently depending on the original molecular structure. Thus, it sometimes gives rise to ions at slightly different m/z, not allowing for a common “benzimidazole core ion type” useful for extracting chromatograms characteristic of multiple TPs. Compared to Omeprazole, these TPs (Table 2) exhibited the main modifications on the pyridine ring while the methoxy-imidazole moiety is retained.

A rearrangement of Omeprazole results in compounds **2**, **3**, **4**, **5**, **11** and **12** losing the sulphur atom. Compounds **7**, **8** and **9** are characterized by the reduction of the sulfoxide group to sulphide. Their detection in the mineral medium of a ready biodegradation test has never been described, but the results are in agreement with what has been described in the literature among the TPs of omeprazole. Compound **2** had already been proposed as a secondary TP of omeprazole by photolysis in water^36^. Compounds **3, 4**, **5**, **6**, **9**, **10**, **11** and **12** were proposed as primary TPs by hydrolysis in water under acidic, basic or neutral conditions as well under oxidative degradative conditions^36^. Compounds **7**, **8** and **10** have been reported as metabolites in human urine and found in wastewater^36,39^. Compounds **9** and **10** have also been described as metabolites in mouse brain and plasma^40^. The EICs of omeprazole and its TPs listed in Table 2 (Figures SI-3–SI-14) and the spectra of the TPs (Figures SI-15–SI-25) are shown in the supplementary information.

### Comparison of the OECD 301F RBT results with metabolism data from human urine and wastewater samples

Results from OECD 301F RBT analysis can be compared with information on human urine metabolism of omeprazole and with data from italian wastewater and surface water sample analysis^38^. The most abundant omeprazole metabolites in urine are 5-hydroxymethyl omeprazole, 5-carboxymethyl omeprazole, 5-O-desmethyl omeprazole and omeprazole sulphone, where omeprazole sulphone was a minor metabolite and omeprazole itself was very low in abundance^38,42^. About seventy different omeprazole metabolites are reported in the literature and detected in urine by mass spectrometry^41^. Boix^38^ elucidated twenty-four urinary omeprazole metabolites making use of UHPLC-qToF, of which nine were found in fifty-two surface water and wastewater samples. Comparing these data with our results, it can be said that four urinary omeprazole metabolites were tentatively identified in our study: the sulphide derivatives TP-7, TP-8, TP-9 and TP-10. Among these, TP-9 (omeprazole sulphide or ufiprazole, the nonenzymatic conversion product of omeprazole) was the most abundant compound formed in our OECD 301F RBT (Table 4). In agreement with Boix^43^ it was also the main compound found in surface water and urban wastewater.

It seems evident that monitoring unchanged omeprazole is not the best strategy to investigate the impact of this widely consumed pharmaceutical in the aquatic ecosystem. Instead, it is recommended to focus the search on omeprazole sulphide (TP-9)^43^ and on omeprazole metabolites excreted in urine.

### Targeted analysis with quantitative and semi-quantitative evaluations

The preliminary quali-quantitative data, obtained from the suspect screening analysis of the mixture from the RBT of “Product A” at T28, evidenced that omeprazole sulphide (TP-9), among all the omeprazole TPs, was the most abundant for its relative area (Fig. 3 and Table SI-3). This data is in agreement with the literature available on omeprazole TP^43^. Therefore, to better evaluate the behaviour of omeprazole and omeprazole sulphide (TP-9) in OECD 301F mineral medium at the end of the RBT, the UHPLC-qToF method used to perform the preliminary quali-quantitative evaluations was validated. The results of the validation proved that the UHPLC-qToF method was accurate, sensitive and precise in quantifying omeprazole and omeprazole sulphide (TP-9) (Table 3).

**Table 3:** Targeted analysis.

Parameters		Results omeprazole	Results omeprazole sulphide (TP-9)
Specificity		The blank sample, OECD 301F mineral medium, did not interfere with the peak	The blank sample, OECD 301F mineral medium, did not interfere with the peak
Range (**)		0.05–0.2 μg/mL	0.005–0.2 μg/mL
Linearity		1–0.005 μg/mL, R^2^ = 0.99	1–0.005 μg/mL, R^2^ = 0.99
Accuracy	Low level recovery	98.5%	107.2%
	Medium level recovery	97.9%	117.4%
	High level recovery	89.8%	106.9%
	Global recovery	95.4%	110.5%
Method Precision		RDS 5.5%	RDS 7%
LoQ		0.005 μg/mL	0.005 μg/mL

Validated method parameters (*).

*performed in a GLP laboratory. **Based on the solution of accuracy test.

The quantitative data of omeprazole and omeprazole sulphide (TP-9) at day 28 of the RBT (T28) are reported in Table 4. Omeprazole concentration resulted 5.4 mg/L at T0 and 1.13 mg/L at T28, revealing that only 20% of the original amount was still present at the end of the RBT. Omeprazole sulphide (TP-9), which was a slight impurity of omeprazole at T0, increases its concentration 4.4-fold and resulted at a concentration of 1.57 mg/L, or the 29% of the original quantity of omeprazole. By summing the quantity of non-biodegraded omeprazole and that transformed into omeprazole sulphide (TP-9), it can be said that 49% of the starting omeprazole is persistent after the 28-day RBT.

**Table 4:** Quantitative data of omeprazole and omeprazole sulphide on day 28th of the RBT^1^.

Compound	Concentration (mg/L)
Omeprazole-1	1.13
Omeprazole sulphide (TP-9)	1.57
Total	2.70

^*1*^Result of the test performed in a GLP test laboratory, where all the activities were managed according to GLP principles. Values are the average of two determinations.

For the other omeprazole TPs a semi-quantitative evaluation was adopted, as reference pure standards were not available. Is not easy to carry out semi-quantitative determinations, especially using LC–MS instruments, due to the different ionization yields of compounds with a different chemical structure^44,45^. In this study, the compounds **6**, **7**, **8** and **10** were semi-quantified (Table 5). These were the most structurally related to omeprazole or omeprazole sulphide (TP-9) and fell within a retention time tolerance of 15%, compared to the retention time of the parent compounds. For the other TPs, however, more appropriate standards for their semi-quantification will have to be identified, in order to produce correct data.

**Table 5:** Semi-quantitative evaluation of omeprazole TPs on day 28th of the RBT^1^.

TP _Number	Compound concentration (mg/L^2^)
Omeprazole **TP-6**	0.25*
Omeprazole **TP-7**	< LoQ (< 0.005)**
Omeprazole **TP-8**	< LoQ (< 0.005)**
Omeprazole **TP-10**	0.01*

^1^Result of the test performed in a GLP test laboratory, where all the activities were managed according to GLP principles. Values are the average of two determinations.

^2^Calculated versus the calibration curve of omeprazole (*) or omeprazole sulphide (TP-9) (**).

Pure omeprazole has been reported to be not RBD^23,25^, with 0.6% degradation^24^. The “not-ready biodegradability” classification has been confirmed at a concentration of 4.4 mg/L (Table 1), with a 2% degradation. Considering the quantitative data of “Product A”, 49% omeprazole is still present at T28 (20% non-biodegraded and 29% transformed to omeprazole sulphide, TP-9). Therefore, omeprazole can be confirmed neither biodegradable in the mixture “Product A”, nor as a pure compound.

## Conclusion

In this manuscript was investigated the output of the ready biodegradability of two commercially available formulations currently used to treat reflux disease and functional dyspepsia, applying the manometric respirometry test (OECD 301F/C.4-D method). “Product A” (containing synthetic derivatives) resulted not readily biodegradable, while “Product B” (containing only natural compounds) resulted fully biodegradable. The samples obtained from RBT were studied by the UHPLC-qToF “all-ion MS/MS” technique to perform untargeted, suspect screening and targeted analysis, in order to follow the behaviour of the two mixtures.

Significant changes in the samples from the beginning of the manometric respirometry test to the 28th day were observed, as a result of the consumption of the products by the microfauna. An incomplete mineralization of “Product A” was evidenced by the untargeted analysis as well as by the suspect screening study. The preliminary data of Omeprazole were also confirmed through targeted quantitative determination, with the method validated according to GLP principles. On the other hand, for “Product B” the untargeted analysis evidenced an almost complete mineralization.

This study confirms that naturally occurring components of complex formulations are more easily biodegradable with respect to non-naturally occurring components; moreover, the use of UHPLC-qToF “all-ion MS/MS” methods offers a very sensitive approach, making it possible to investigate the output of uncomplete biodegradations in great depth. In these latter cases, new mixtures of compounds are produced with an unpredictable impact on the environment. Furthermore, this approach is ideal for studying drug metabolites and their transformation products, which is an issue of general interest for determining the environmental contamination of drugs.

In conclusion, accurate analysis and characterization will strongly improve the quality of the biodegradability evaluation of formulated commercial products, offering considerable advantages in controlling and preventing potential environmental hazards.

### Supplementary Information

Supplementary Information.

## Data Availability

All data used are in the manuscript and its supplements. Original spectral data can be provided upon reasonable request contacting Dr. Luisa Mattoli (LMattoli@aboca.it).
