# Publisher Correction: Suspect screening analysis to improve untargeted and targeted UHPLC-qToF approaches: the biodegradability of a proton pump inhibitor medicine and a natural medical device

**DOI:** 10.1038/s41598-024-53924-1

**Published:** 2024-03-07

**Authors:** Luisa Mattoli, Giacomo Proietti, Giada Fodaroni, Claudio Marzio Quintiero, Michela Burico, Mattia Gianni, Emiliano Giovagnoni, Valentino Mercati, Claudio Santi

**Affiliations:** 1 Metabolomics and Analytical Sciences, Aboca SpA, Sansepolcro, AR Italy; 2 Group of Catalysis, Synthesis and Organic Green Chemistry, Department of Pharmaceutical Sciences, University of Perugia, Via del Liceo 1, 06123 Perugia, Italy; 3 Centro di Eccellenza Materiali Innovativi Nanostrutturati (CEMIN), University of Perugia, Via Elce di Sotto 8, 06123 Perugia, Italy

Correction to:* Scientific Reports* 10.1038/s41598-023-49948-8, published online 02 January 2024

The Competing interests section in the original version of this Article was incorrect.

“The authors declare no competing interests.”

now reads:

“The authors listed below report the following details of affiliation or involvement in an organization or entity with a financial interest in the subject matter or materials discussed in this manuscript.

LM, GP, GF, CMQ, GF, MB, GM are employed in Aboca who produce and commercialize the product indicated in the manuscript as B. VM is Honorary President of Aboca SPA. CS is the scientific responsible of a project related to the topic of the present manuscript and committed by Aboca to C.E.M.I.N (UniPG). Product A is a generic drug containing Omeprazole, while “Product B”, Neobianacid, is a Medical device made of substances. Both products are indicated for the treatment reflux diseases and functional dyspepsia.”

The original Article has been corrected.

