# Supplementary material for: Suspect screening analysis to improve untargeted and targeted UHPLC-qToF approaches: the biodegradability of a proton pump inhibitor medicine and a natural medical device
Source: Sci Rep. 2024 Jan 2;14:51. doi: 10.1038/s41598-023-49948-8 (PMC10761695; doi:10.1038/s41598-023-49948-8)
Supplement: Supplementary file 1 — Supplementary Information. [file 41598_2023_49948_MOESM1_ESM.docx]

**SUPPORTING INFORMATION**

**Suspect screening analysis to improve untargeted and targeted UHPLC-qToF approaches: the biodegradability of a Proton Pump Inhibitor medicine and a natural medical device**

Luisa Mattoli^a‡^, Giacomo Proietti^a‡^, Giada Fodaroni^a^, Claudio Marzio Quintiero^a^, Michela Burico^a^, Mattia Gianni^a^, Emiliano Giovagnoni^a^, Valentino Mercati^a^, Claudio Santi*^b,c^

^a^ Metabolomics & Analytical Sciences, Aboca SpA, Sansepolcro (AR), Italy.

^b^ Group of Catalysis, Synthesis and Organic Green Chemistry – Department of Pharmaceutical Sciences University of Perugia, Via del Liceo 1, 06123 Perugia, Italy.

^c^ Centro di Eccellenza Materiali Innovativi Nanostrutturati (CEMIN) University of Perugia, Via Elce di Sotto 8, 06123, Perugia, Italy.

**CONTENT**

[**Table SI-1. Elution gradient.**](#_Toc152239371) 1

[**Table SI-2. MS parameters** 3](#_Toc152239372)

[**Table SI-3.** Relative area % evaluation of Omeprazole and its TPs at day 28th of the ready biodegradability test. 4](#_Toc152239373)

[**Figure SI-1_ “Product A” and “Product B” Hotelling T2 and dMod X** 5](#_Toc152239374)

[**Figure SI-2_ OPLS of “Product A”: Permutation test of the three difference classes and the corresponding Loading plot (pqcorr_plot).** 6](#_Toc152239375)

[**Figure_SI-3_omeprazole_1**, m/z 346.1221_EIC of fragment at T2_ EIC at m/z 198.0583 (dark line), and at m/z 151.0991 (pink line), m/z 149.0709 (orange line) m/z 136.0757 (blue line). 7](#_Toc152239376)

[**Figure_SI-4_omeprazole TP_2**, m/z 182.0812_EIC of fragment at T28_EIC at m/z 154.0863 (dark line) and at m/z 136.0757 (brown line). 8](#_Toc152239377)

[**Figure_SI-5_omeprazole TP_3**, m/z 165.0658_EIC of fragment at T28_EIC at m/z 150.0423 (orange line). 8](#_Toc152239378)

[**Figure_SI-6_omeprazole TP_4**, m/z 298.1560_EIC of fragment at T28_EIC at m/z 283.1315 (violet line), m/z 268.1081 (blue line), m/z 150.0913 (green line), m/z 147.0552 (red line) and at m/z 120.0808 (black line). 9](#_Toc152239379)

[**Figure_SI-7_omeprazole TP_5**, m/z 314.1135_EIC of fragment at T28_EIC at m/z 296.1029 (black line), m/z 270.1237 (pink line), m/z 268.1080 (orange line), 255.1002 m/z (light blue line), m/z 122.0600 (green line). 10](#_Toc152239380)

[**Figure_SI-8_omeprazole TP_6**, m/z 316.1114_EIC of fragment at T28_EIC at m/z 301.0879 (green line), m/z 284.0852 (brown line), 168.0477 (violet line), m/z 149.0709 (red line) and at m/z 136.0757 (dark green line). 11](#_Toc152239381)

[**Figure_SI-9_omeprazole TP_7**, m/z 360.1018_EIC of fragment at T28_EIC at m/z 327.1219 (dark line), m/z 212.0381 (violet line) and at m/z 180.0661 (orange line). 12](#_Toc152239382)

[**Figure_SI-10_omeprazole TP_8**, m/z 346.1225_EIC of fragment at T28_EIC at m/z 313.1426 (violet line), 198.0589 (green line), m/z 152.0712 (blue line) and at m/z 138.0919 (orange line). 13](#_Toc152239383)

[**Figure_SI-11_omeprazole TP_9**, m/z 330.1270_EIC of fragment at T28_EIC at m/z 297.1471 (violet line), m/z 267.1366 (black line), m/z 182.0634 (red line), m/z 166.0321 (dark green line), m/z 150.0913 (blue line), m/z 149.0709 (purple line), m/z 136.0757 (brown line), m/z 120.0807 (green line). 15](#_Toc152239384)

[**Figure_SI-12_omeprazole TP_10**, m/z 362.1169_EIC of fragment at T28_EIC at m/z 195.0222 (black line), m/z 166.0868 (red line), and m/z 150.0913 (green line), 149.0709 (blue line), 120.0807 (violet line). 16](#_Toc152239385)

[**Figure_SI-13_omeprazole TP_11**, m/z 284.1393_EIC of fragment at T28_EIC at m/z 269.1158 (green line), m/z 268.1080 (pink line), m/z 254.0924 (orange line), m/z 147.0552 (pink line) and at m/z 136.0757 (black line). 17](#_Toc152239386)

[**Figure_SI-14_omeprazole TP_12**, m/z 312.1342_ EIC of fragment at T28_EIC at m/z 297.1107 (green line), m/z 269.1158 (blue line) and at m/z 252.1131 (violet line). 18](#_Toc152239387)

[**Figure SI-15** **Spectrum of Structure omeprazole TP_2**. Accurate mass spectrum at m/z 182.0816 and 7.062 min (left) then the corresponding MS/MS fragmentation pattern at 30eV (right) with ions at m/z 154.0865 and 136.0760. 19](#_Toc152239388)

[**Figure SI-16** **Spectrum of Structure omeprazole TP_3**. Accurate mass spectrum at m/z 165.0661 and 10.430 min (left) then the corresponding MS/MS fragmentation pattern at 30eV (right) with ions at m/z 150.0425 and 122.0480. 19](#_Toc152239389)

[**Figure SI-17** **Spectrum of Structure omeprazole TP_4.** Accurate mass spectrum at m/z 298.1555 and 12.579 min (left) then the corresponding MS/MS fragmentation pattern at 30eV (right) with ions at m/z 283.1315, 268.1081, 147.0556, 150.0914 and 120.0810. 20](#_Toc152239390)

[**Figure SI-18** **Spectrum of Structure omeprazole TP_5.** Accurate mass spectrum at m/z 314.1139 and 12.630 and 12.839 min (left) then the corresponding MS/MS fragmentation pattern at 30eV (right) with ions at m/z 296.1030, 270.1238, 268.1084, 255.1006 and 122.0604. 21](#_Toc152239391)

[**Figure SI-19** **Spectrum of Structure omeprazole TP_6.** Accurate mass spectrum at m/z 316.1128 and 13.104 min (left) then the corresponding MS/MS fragmentation pattern at 30eV (right) with ions at m/z 301.0878, 284.0853, 168.0483, 149.0713, 136.0762. 22](#_Toc152239392)

[**Figure SI-20** **Spectrum of Structure omeprazole TP_7.** Accurate mass spectrum at m/z 360.1015 and 13.227 min (left) then the corresponding MS/MS fragmentation pattern at 30eV (right) with ions at m/z 327.1205, 212.0380, 180.0664. 23](#_Toc152239393)

[**Figure SI-21** **Spectrum of Structure omeprazole TP_8.** Accurate mass spectrum at m/z 346.1219 and 13.290 min (left) then the corresponding MS/MS fragmentation pattern at 30eV (right) with ions at m/z 313.1414, 198.0588, 152.0708, 138.0918. 24](#_Toc152239394)

[**Figure SI-22** **Spectrum of Structure omeprazole TP_9.** Accurate mass spectrum at m/z 330.1283 and 14.711 min (left) then the corresponding MS/MS fragmentation pattern at 30eV (right) with ions at m/z 297.1477, 267.1366, 182.0639, 166.0320, 150.0920, 149.0717, 136.0762, 120.0815. 25](#_Toc152239395)

[**Figure SI-23** **Spectrum of Structure omeprazole TP_10.** Accurate mass spectrum at m/z 362.1171 and 15.659 min (left) then the corresponding MS/MS fragmentation pattern at 30eV (right) with ions at m/z 195.0218, 166.0872, 150.0917, 149.0718, 120.0811. 26](#_Toc152239396)

[**Figure SI-24** **Spectrum of Structure omeprazole TP_11.** Accurate mass spectrum at m/z 284.1397 and 15.744 min (left) then the corresponding MS/MS fragmentation pattern at 30eV (right) with ions at m/z 269.1163, 268.1076, 254.0930, 147.0555, 136.0716. 27](#_Toc152239397)

[**Figure SI-25** **Spectrum of Structure omeprazole TP_12.** Accurate mass spectrum at m/z 312.1352 and 16.218 min (left) then the corresponding MS/MS fragmentation pattern at 30eV (right) with ions at m/z 297.1107, 269.1161, 252.1126. 28](#_Toc152239398)

## **Table SI-1. Elution gradient.**

|  | **Product A** | | **Product B** | |
| --- | --- | --- | --- | --- |
| **Time (min)** | **A%** | **B%** | **A%** | **B%** |
| 0,0 | 99 | 1 | 99 | 1 |
| 1,0 | - | - | 99 | 1 |
| 2,0 | 99 | 1 | 75 | 25 |
| 5,0 | 90 | 10 | - | - |
| 10,0 | 75 | 25 | 50 | 50 |
| 11,0 | - | - | 50 | 50 |
| 15,0 | 50 | 50 | 25 | 75 |
| 16,0 | 25 | 75 | - | - |
| 17,0 | - | - | 15 | 85 |
| 18,0 | 10 | 90 | - | - |
| 19,0 | - | - | 1 | 99 |
| 19,5 | - | - | 1 | 99 |
| 20,0 | 10 | 90 | - | - |
| 21,0 | 1 | 99 | 99 | 1 |
| 24,0 | 1 | 99 | 99 | 1 |

## **Table SI-2. MS parameters**

| **Instrumental parameters** | **ESI-** | **ESI+** |
| --- | --- | --- |
| Gas temperature (°C) | 325 | 325 |
| Gas flow (L min-1) | 11 | 11 |
| Nebulizer (psig) | 35 | 35 |
| Sheat gas temperature (°C) | 350 | 350 |
| Sheat gas flow (L min-1) | 12 | 12 |
| VCap | 3500 | 4000 |
| Nozzle voltage (V) | 0 | 1500 |
| Fragmentor | 100 | 100 |
| Skimmer | 65 | 65 |
| Octopole RF Peak | 750 | 750 |

## **Table SI-3.** Relative area % evaluation of Omeprazole and its TPs at day 28th of the ready biodegradability test.

| TP_Number | Relative Area %* |
| --- | --- |
| Omeprazole _1 | 0.9 |
| Omeprazole TP _2 | 17.8 |
| Omeprazole TP _3 | 3.7 |
| Omeprazole TP _4 | 11.7 |
| Omeprazole TP _5 | 2.3 |
| Omeprazole TP _6 | 4.3 |
| Omeprazole TP _7 | 0.1 |
| Omeprazole TP _8 | 0.2 |
| Omeprazole TP _9 (Omeprazole sulphide) | 56.3 |
| Omeprazole TP *_*10 | 0.5 |
| Omeprazole TP _11 | 1.9 |
| Omeprazole TP _12 | 0.3 |
|  |  |

**Result of the test performed in an ISO 17025 certified laboratory, where all the activities were managed according to ISO17025 principles.*

*Relative area % evaluation. Area % was determined at T28 considering the omeprazole area counts and that of the TPs reported in Table 2, which were divided by the internal standard (sulfadimethoxine-d6) area count. Then, all the corrected values were summed to get a total area. The percentage of the area of each compound at T28 is calculated by dividing each corrected area by the total area and multiplied by 100.*

| 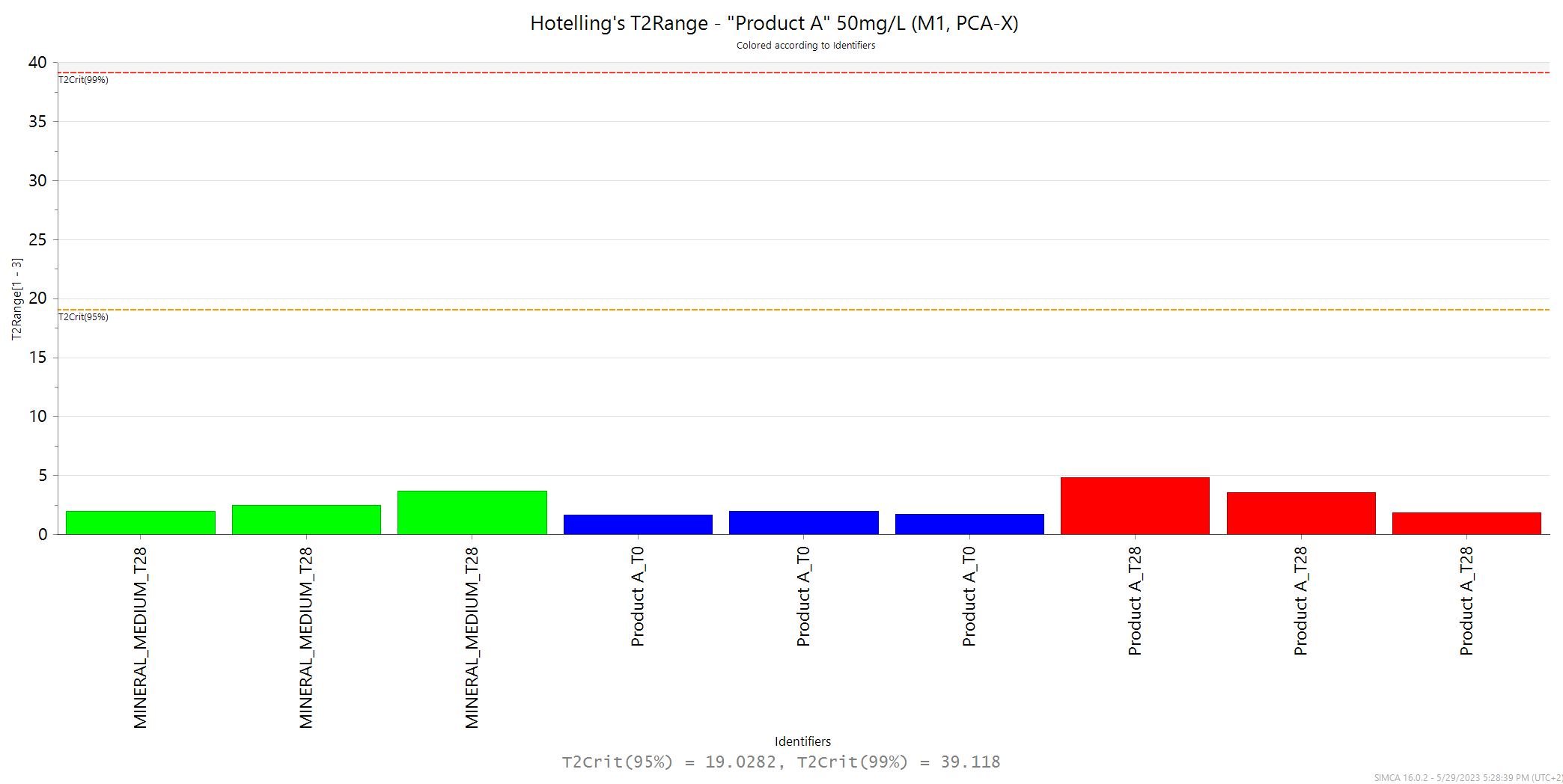 | 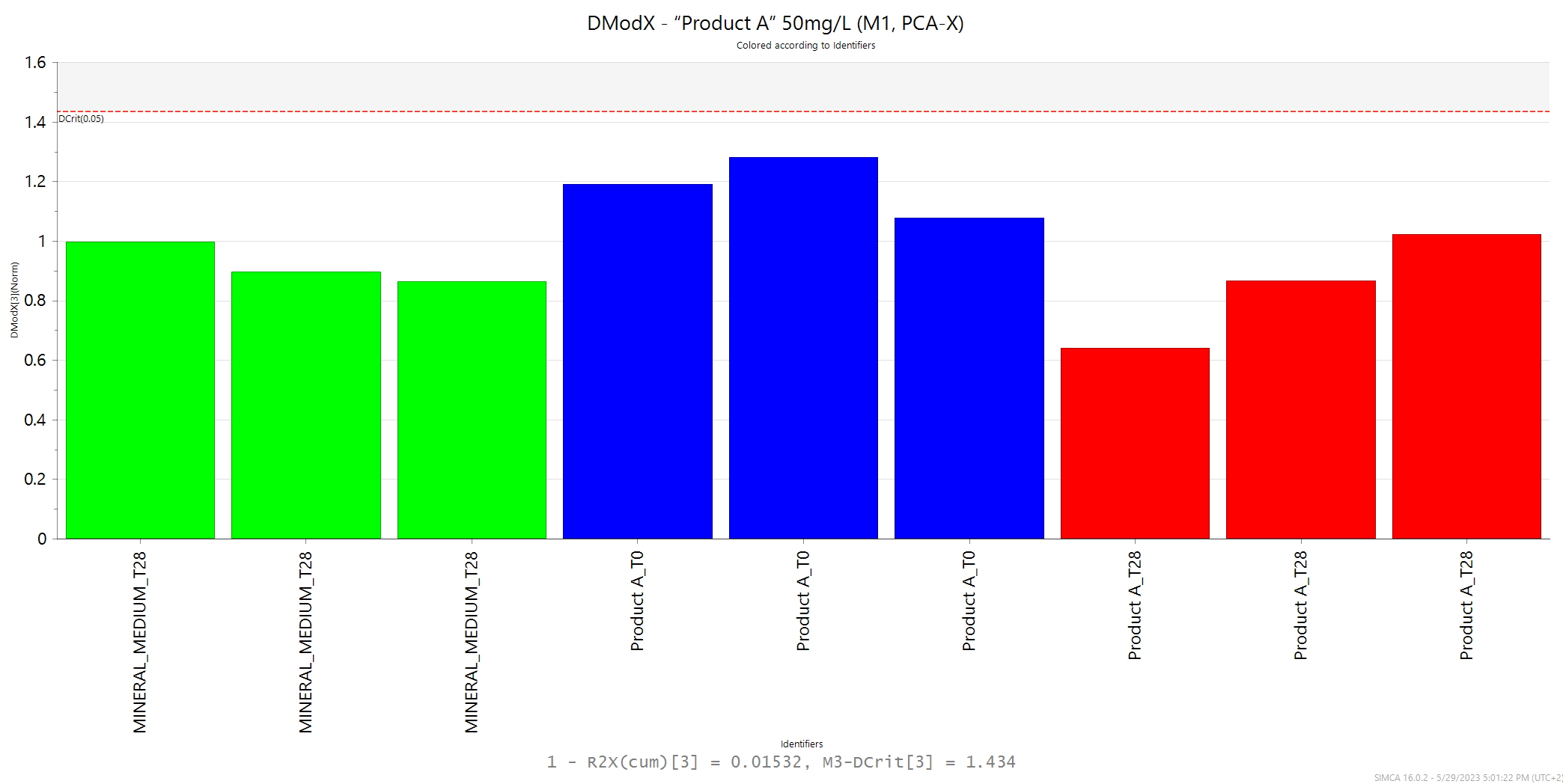 |
| --- | --- |
| “Product A”_50 mg/L_Hotelling T^2^ | “Product A”_50 mg/L_ dMod X |
| 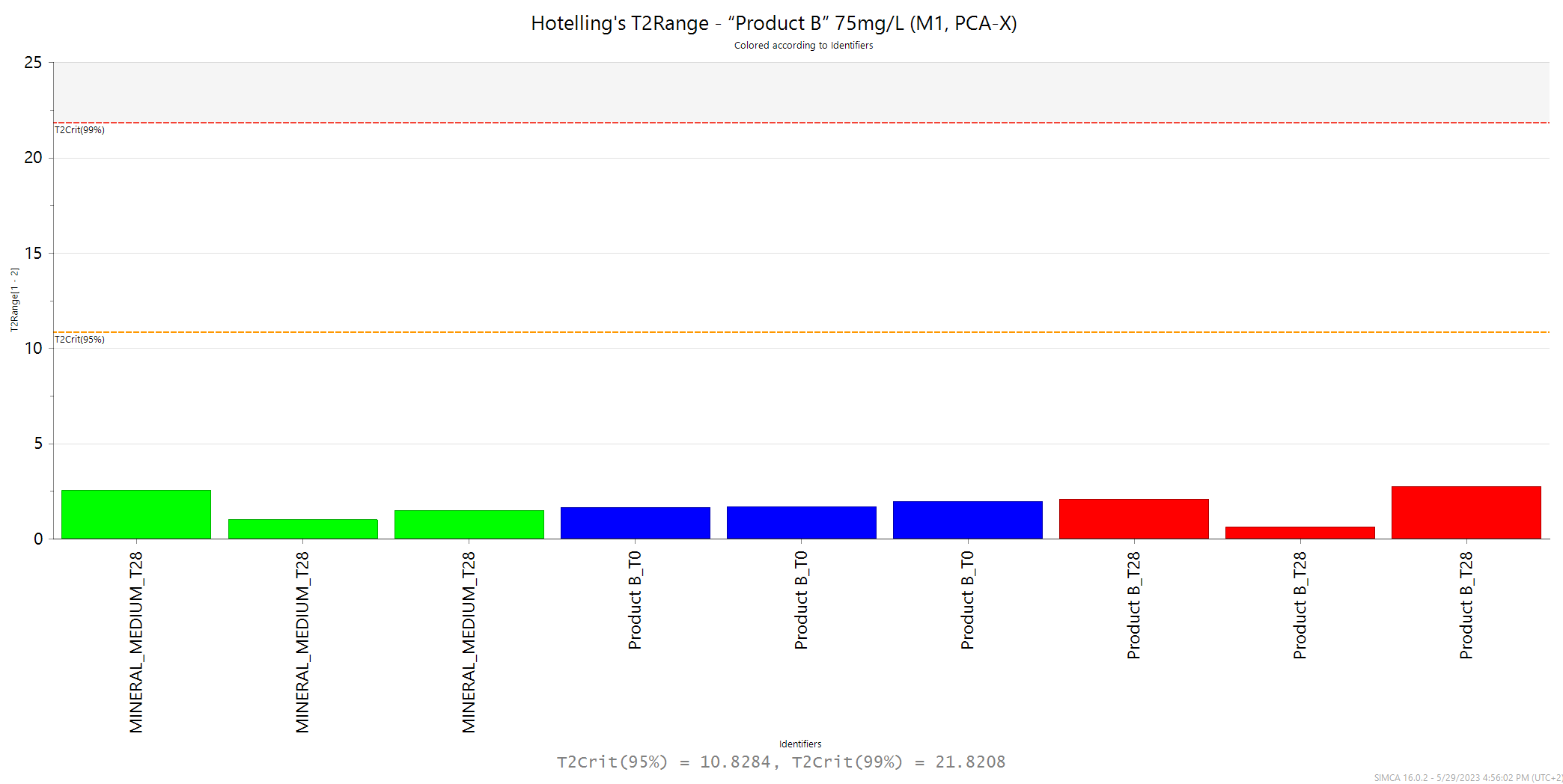 | 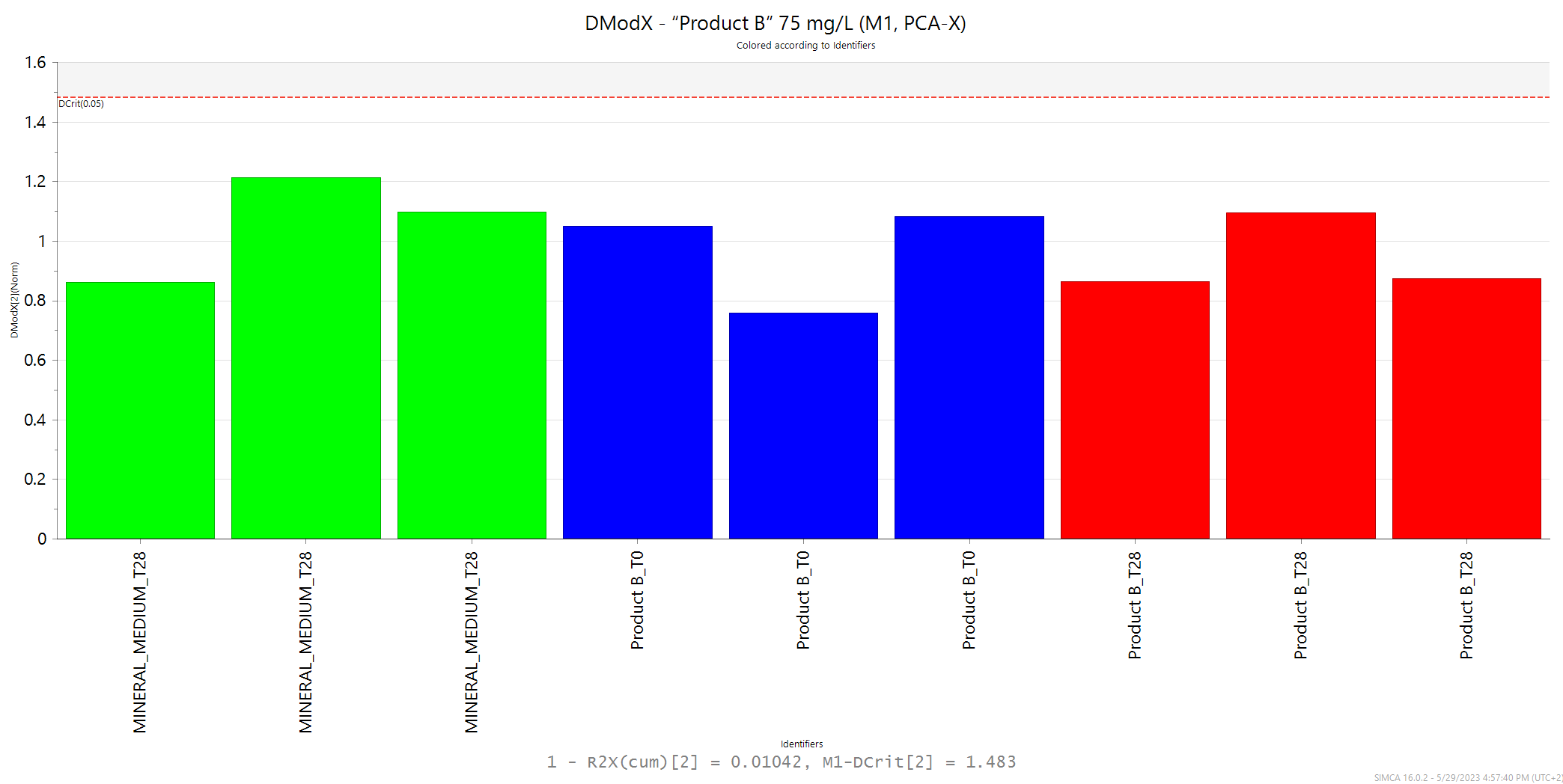 |
| “Product B” _75 mg/L_ Hotelling T^2^ | “Product B”_75 mg/L_ dMod X |

## **Figure SI-1_ “Product A” and “Product B” Hotelling T2 and dMod X**

| 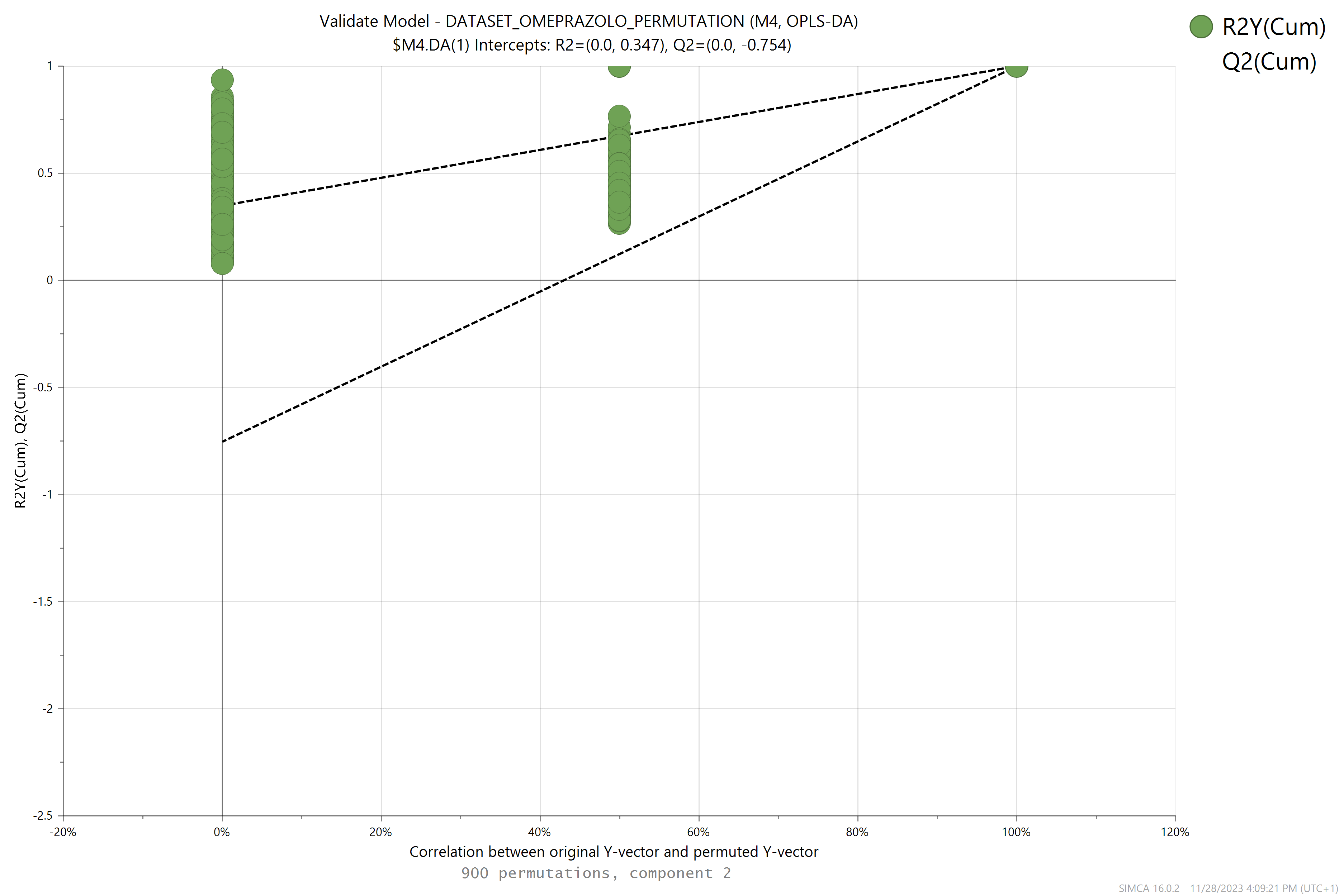 | 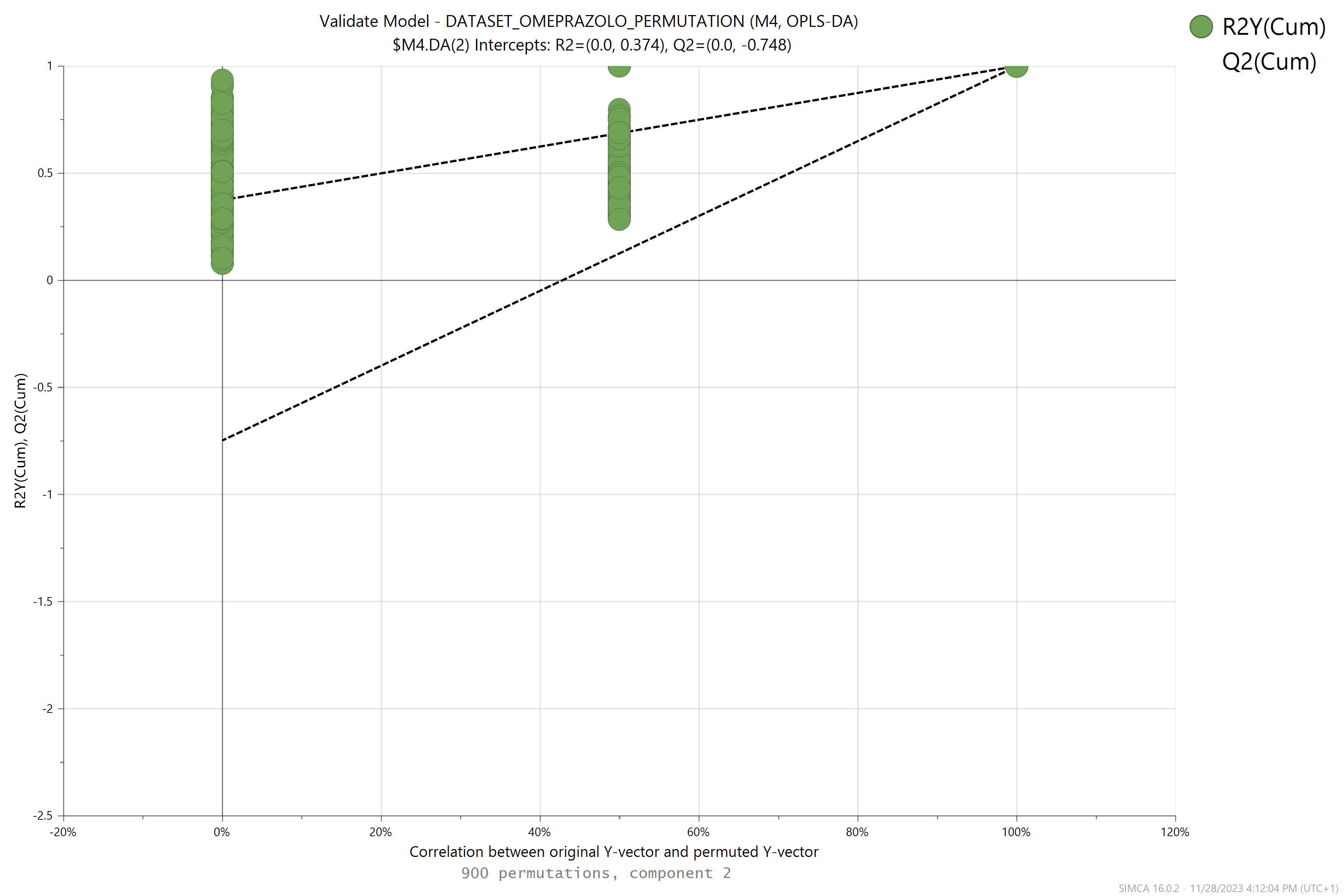 |
| --- | --- |
| Permutation test of DA1 class: mineral medium | Permutation test of DA2 class: Product A_Omeprazole at T0 |
| 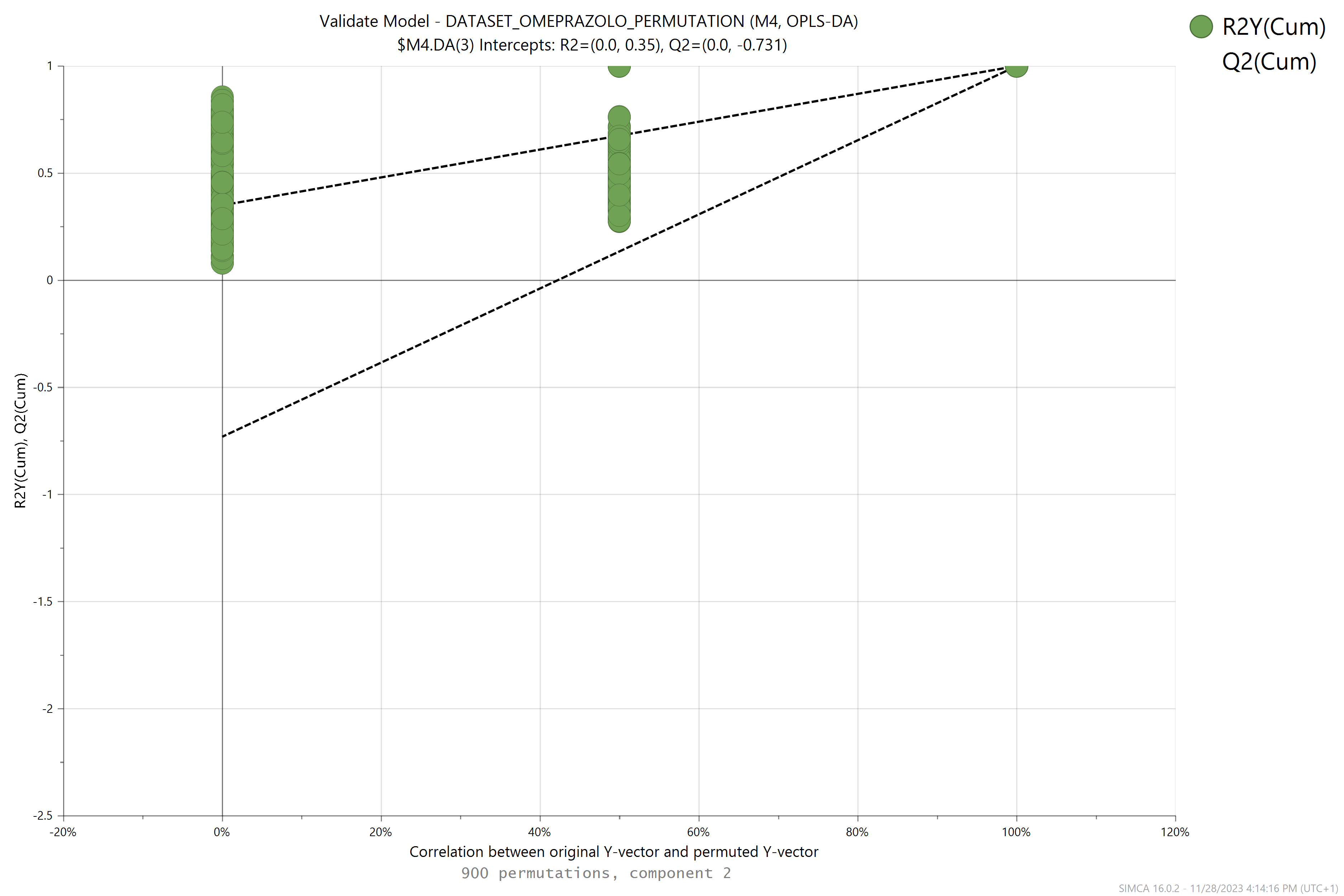 |  |
| Permutation test of DA3 class: Product A_omeprazole at T28 |  |
| 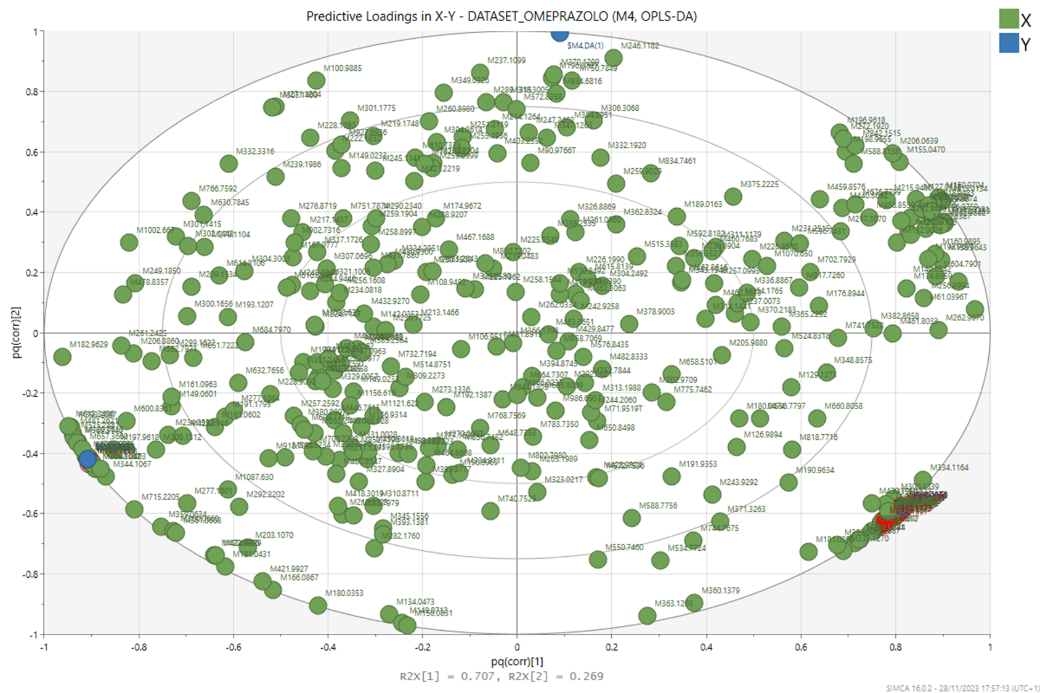  Omeprazole at T28  Omeprazole at T0  Mineral medium at T28 | |
| Product A_Omeprazole_Loading plot (pqcorr_plot) | |

## **Figure SI-2_ OPLS of “Product A”: Permutation test of the three difference classes and the corresponding Loading plot (pqcorr_plot).**

**The extract ion chromatogram (EIC) of Omeprazole (1) fragment ions at T0 and its TPs (2-12) fragment ions after 28 days (Figure SI-3-Figure SI-14).**

| *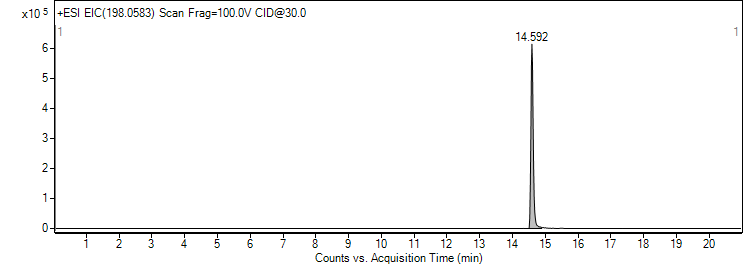*  *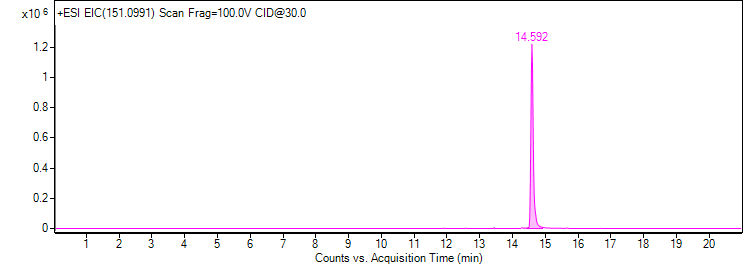*  *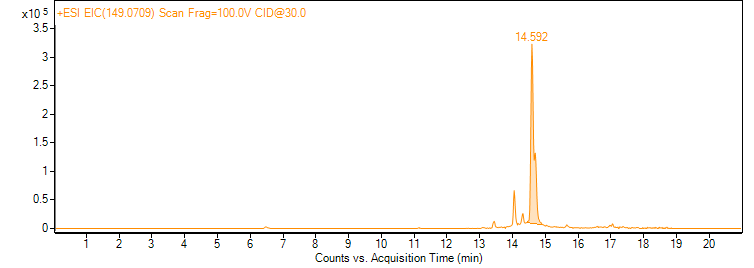*  *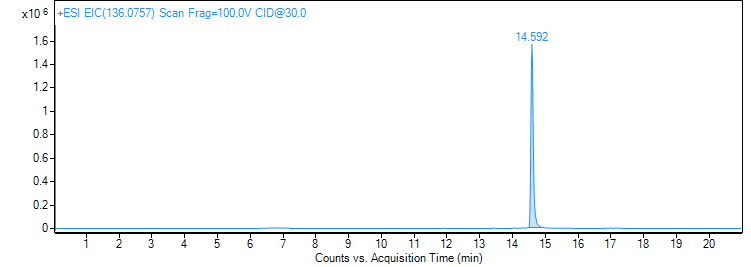* |
| --- |

## **Figure_SI-3_Omeprazole_1**, m/z 346.1221_EIC of fragment at T2_ EIC at m/z 198.0583 (dark line), and at m/z 151.0991 (pink line), m/z 149.0709 (orange line) m/z 136.0757 (blue line).

| *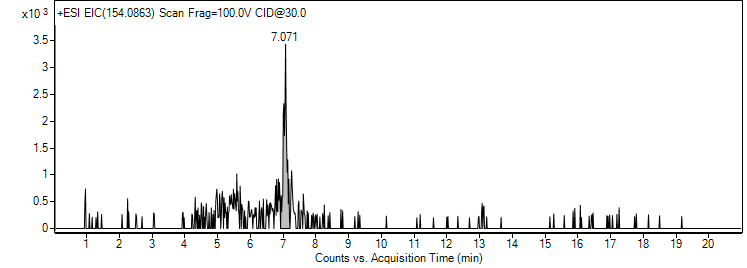*  *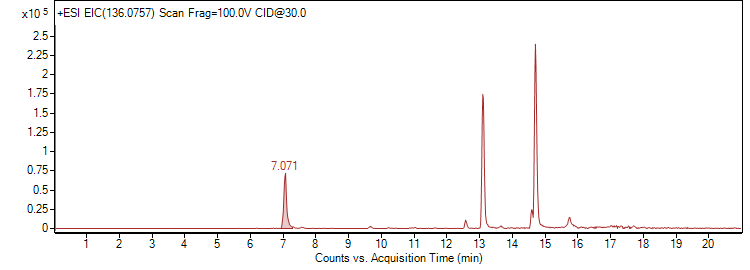* |
| --- |

## **Figure_SI-4_Omeprazole TP_2**, m/z 182.0812_EIC of fragment at T28_EIC at m/z 154.0863 (dark line) and at m/z 136.0757 (brown line).

| *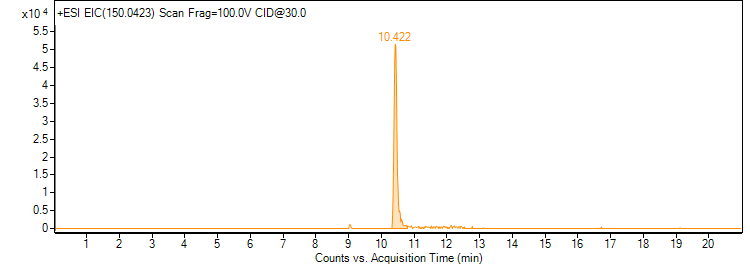* |
| --- |

## **Figure_SI-5_Omeprazole TP_3**, m/z 165.0658_EIC of fragment at T28_EIC at m/z 150.0423 (orange line).

| *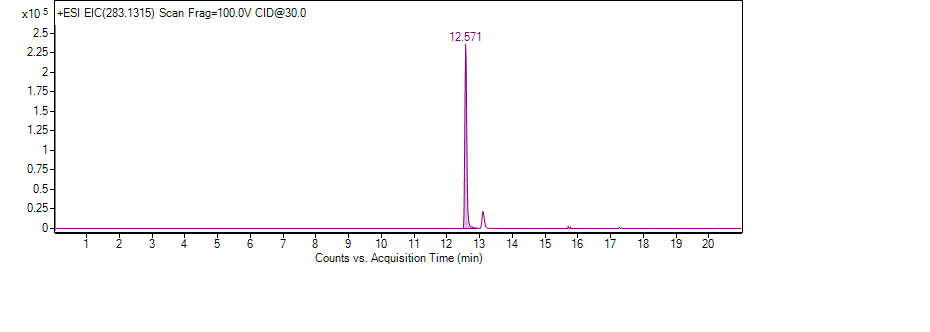*  *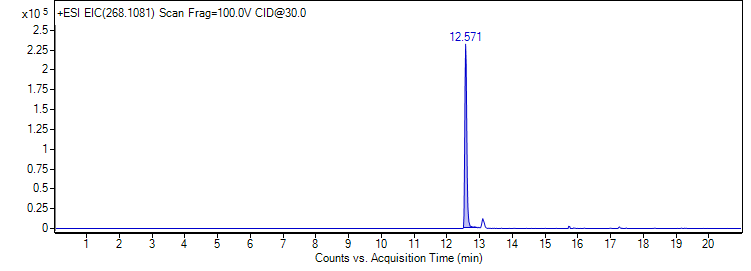*  *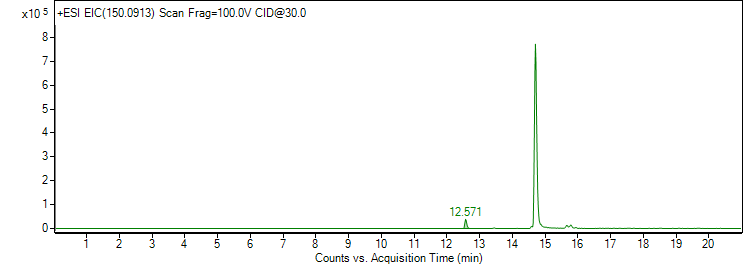*  *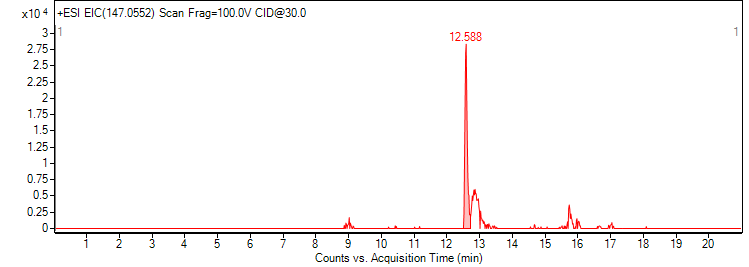*  *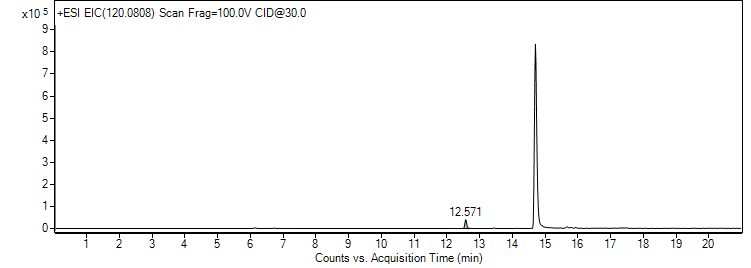* |
| --- |

## **Figure_SI-6_Omeprazole TP_4**, m/z 298.1560_EIC of fragment at T28_EIC at m/z 283.1315 (violet line), m/z 268.1081 (blue line), m/z 150.0913 (green line), m/z 147.0552 (red line) and at m/z 120.0808 (black line).

| *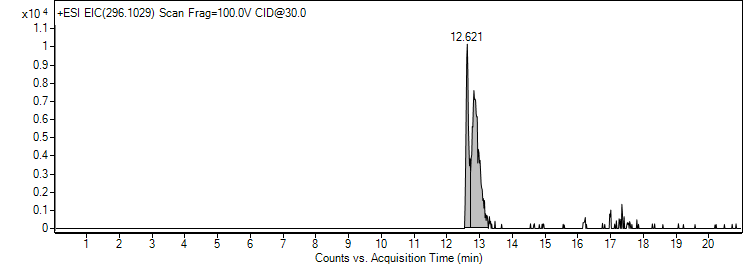*  *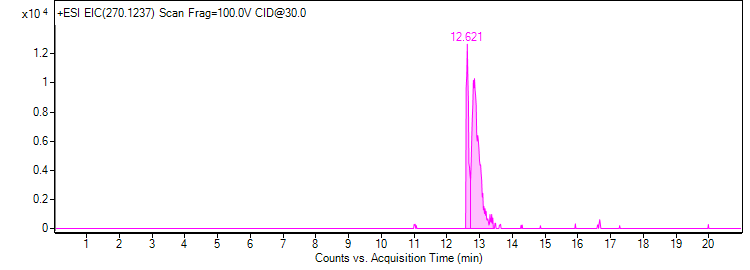*  *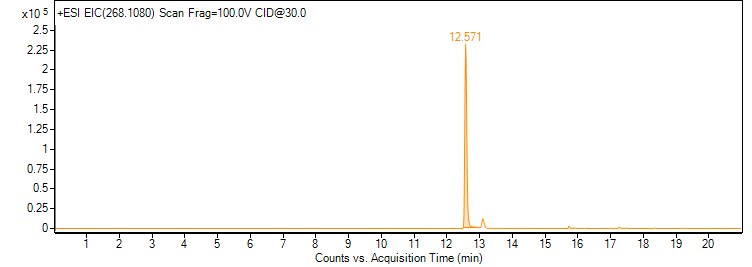*  *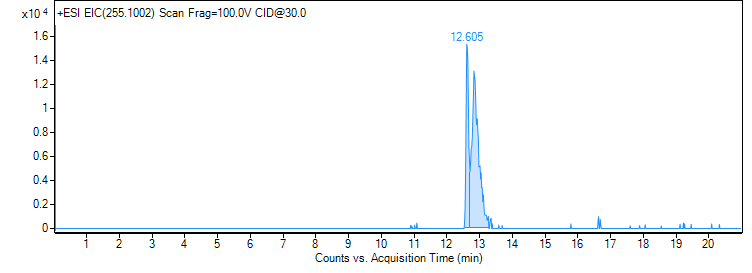*  *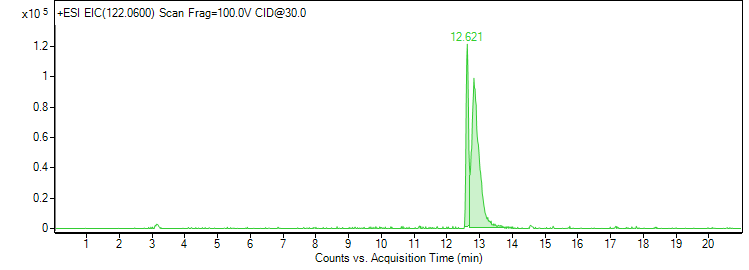* |
| --- |

## **Figure_SI-7_Omeprazole TP_5**, m/z 314.1135_EIC of fragment at T28_EIC at m/z 296.1029 (black line), m/z 270.1237 (pink line), m/z 268.1080 (orange line), 255.1002 m/z (light blue line), m/z 122.0600 (green line).

| *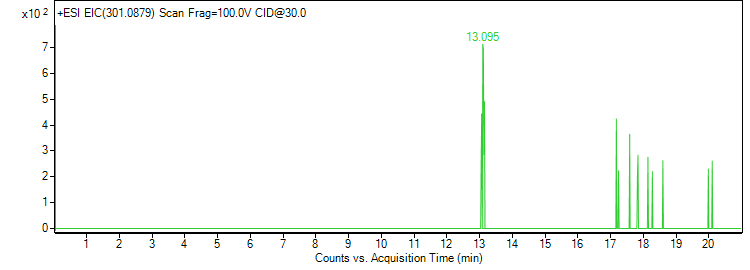*  *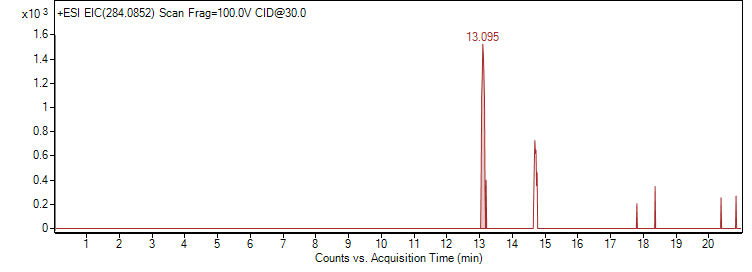*  *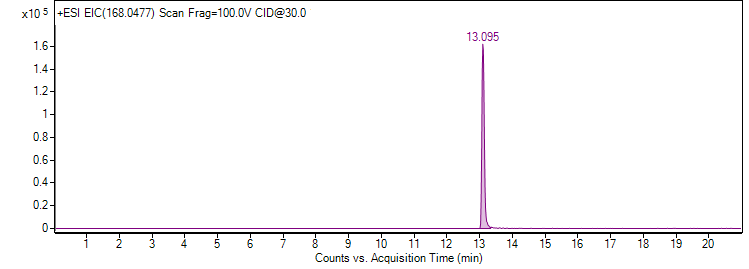*  *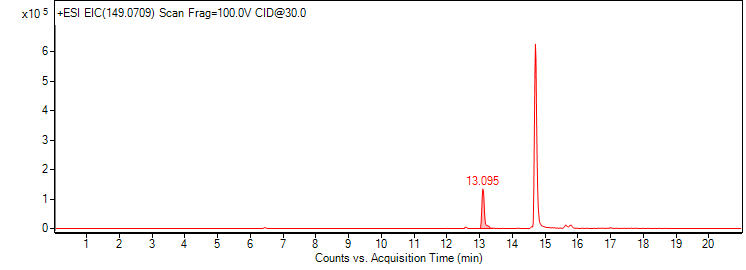*  *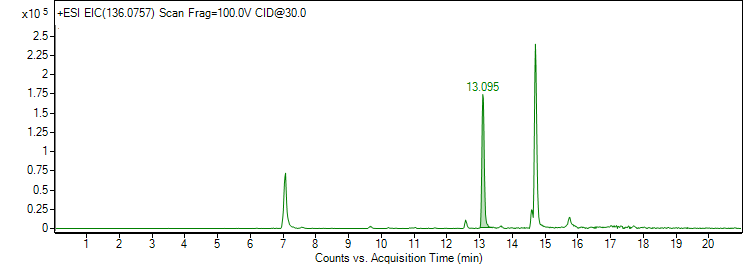* |
| --- |

## **Figure_SI-8_Omeprazole TP_6**, m/z 316.1114_EIC of fragment at T28_EIC at m/z 301.0879 (green line), m/z 284.0852 (brown line), 168.0477 (violet line), m/z 149.0709 (red line) and at m/z 136.0757 (dark green line).

| *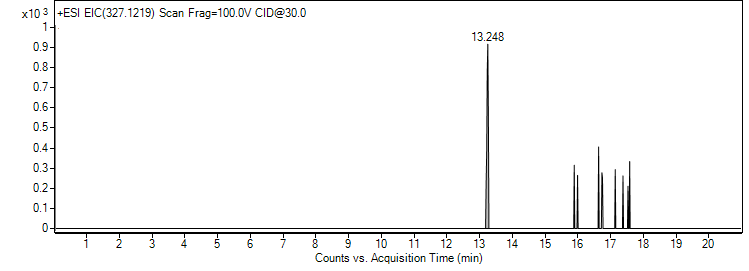*  *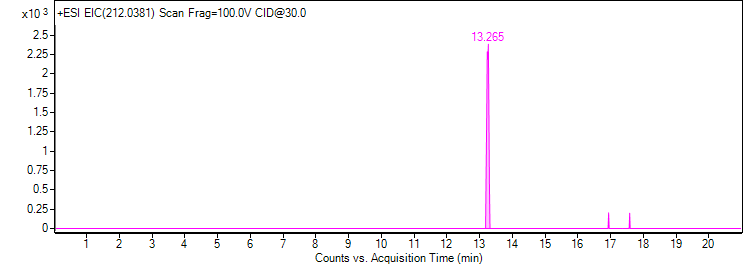*  *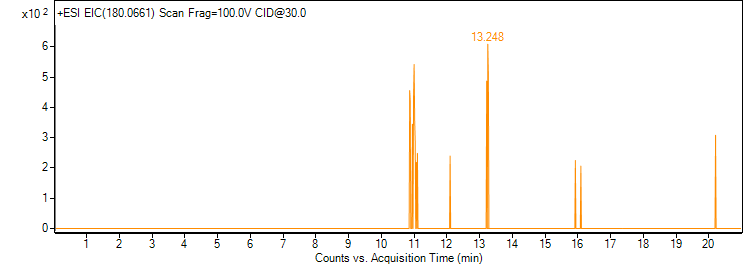* |
| --- |

## **Figure_SI-9_Omeprazole TP_7**, m/z 360.1018_EIC of fragment at T28_EIC at m/z 327.1219 (dark line), m/z 212.0381 (violet line) and at m/z 180.0661 (orange line).

| *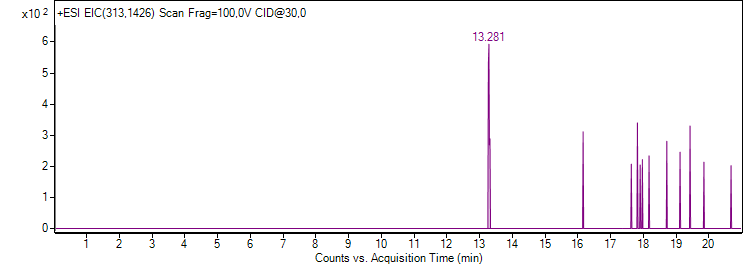*  *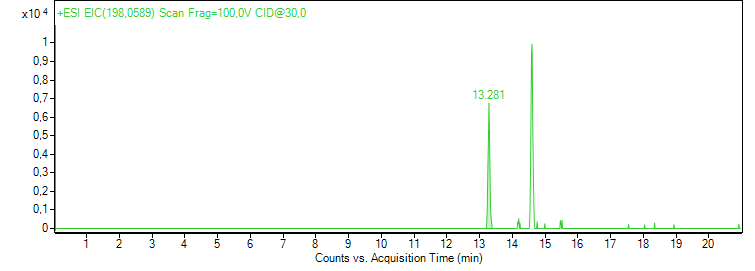*  *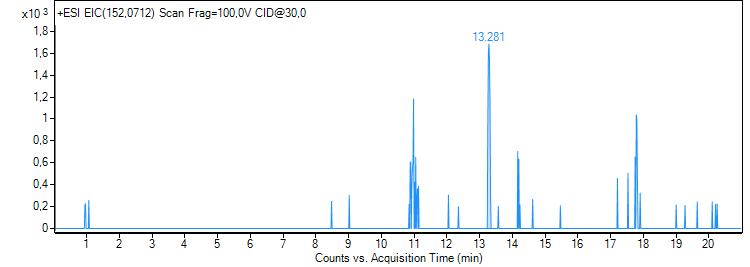*  *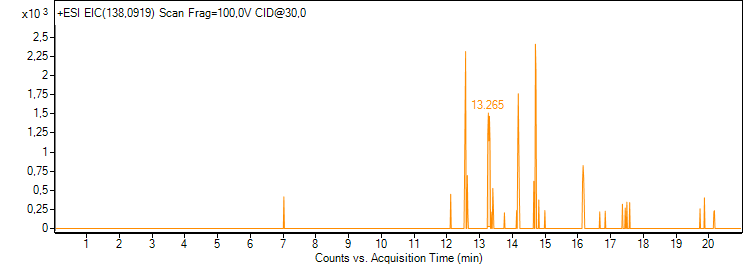* |
| --- |

## **Figure_SI-10_Omeprazole TP_8**, m/z 346.1225_EIC of fragment at T28_EIC at m/z 313.1426 (violet line), 198.0589 (green line), m/z 152.0712 (blue line) and at m/z 138.0919 (orange line).

| *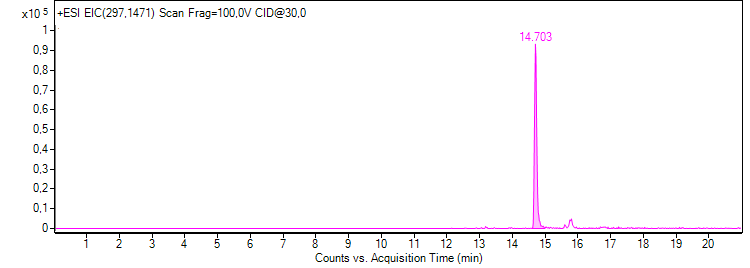*  *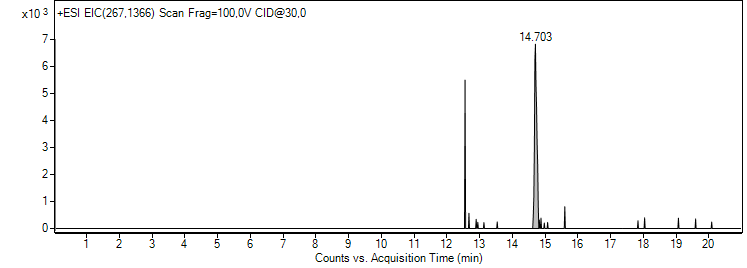*  *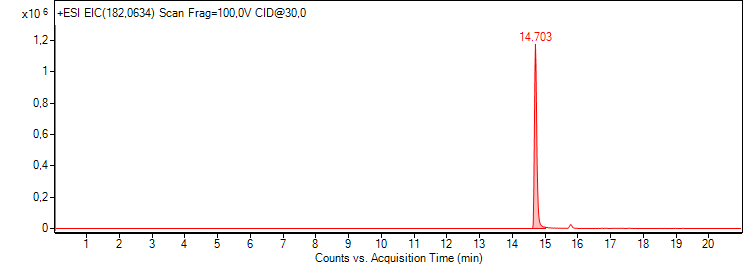*  *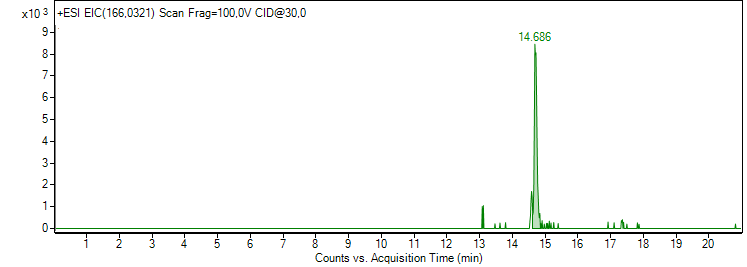*  *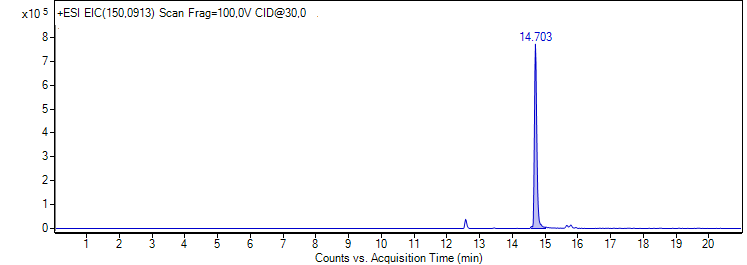*  *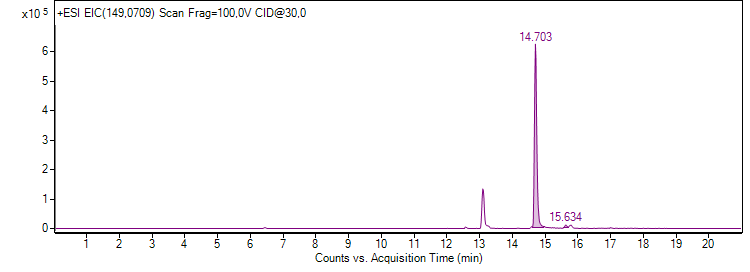*  *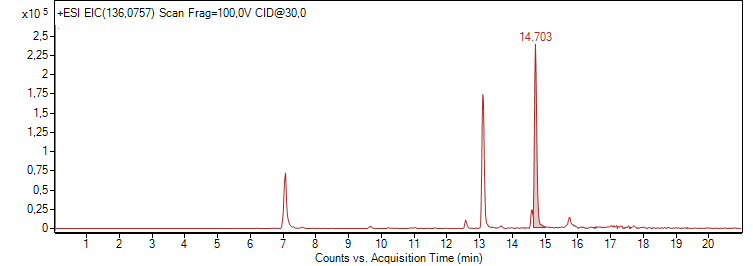*  *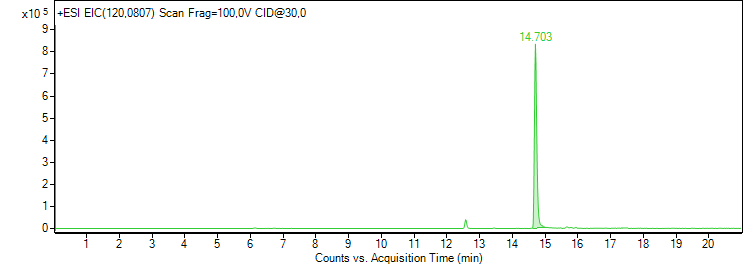* |
| --- |

## **Figure_SI-11_Omeprazole TP_9**, m/z 330.1270_EIC of fragment at T28_EIC at m/z 297.1471 (violet line), m/z 267.1366 (black line), m/z 182.0634 (red line), m/z 166.0321 (dark green line), m/z 150.0913 (blue line), m/z 149.0709 (purple line), m/z 136.0757 (brown line), m/z 120.0807 (green line).

| *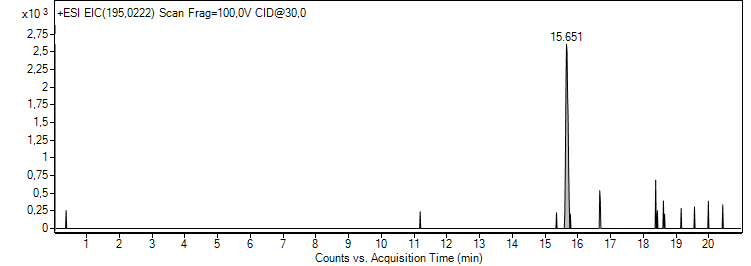*  *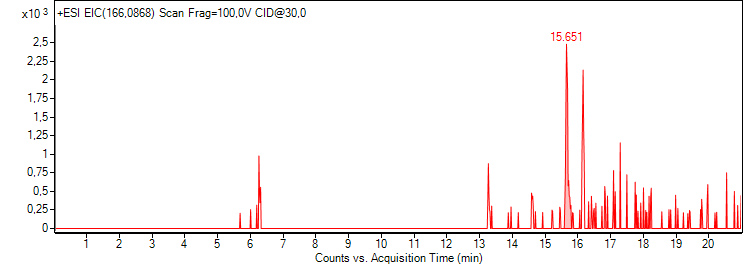*  *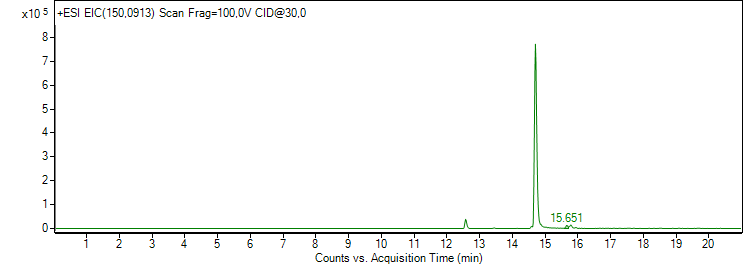*  *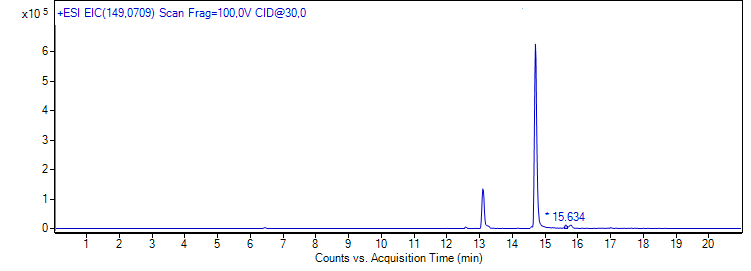*  *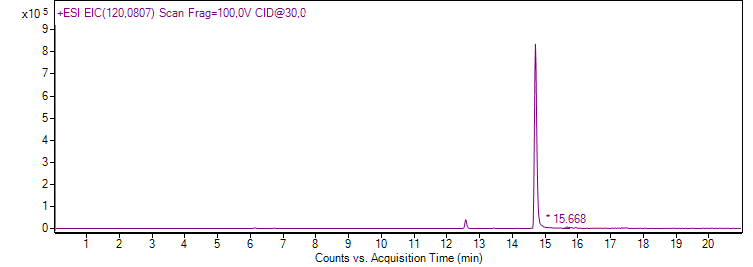* |
| --- |

## **Figure_SI-12_Omeprazole TP_10**, m/z 362.1169_EIC of fragment at T28_EIC at m/z 195.0222 (black line), m/z 166.0868 (red line), and m/z 150.0913 (green line), 149.0709 (blue line), 120.0807 (violet line).

| *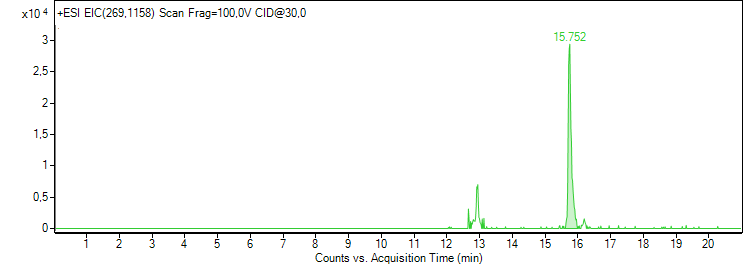*  *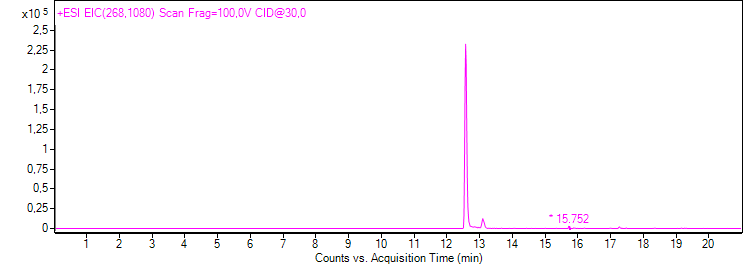*  *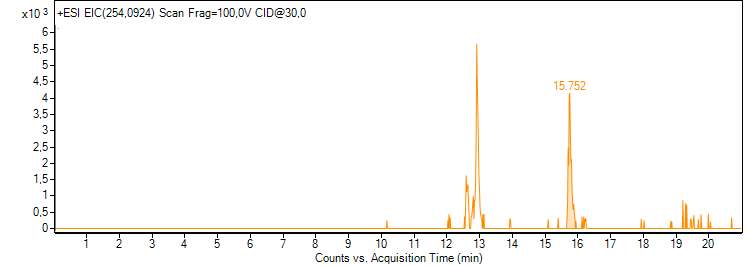*  *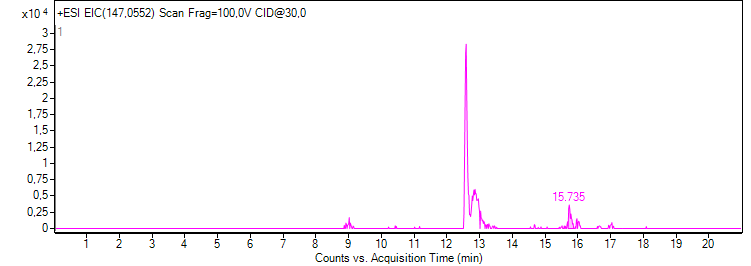*  *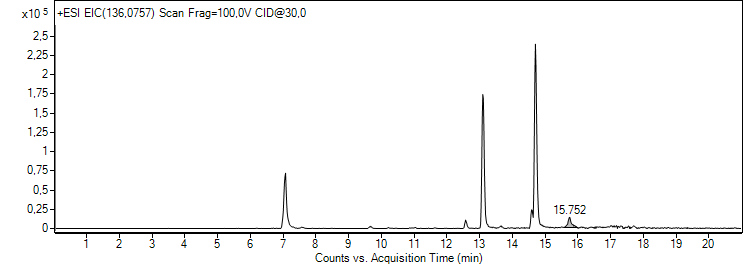* |
| --- |

## **Figure_SI-13_Omeprazole TP_11**, m/z 284.1393_EIC of fragment at T28_EIC at m/z 269.1158 (green line), m/z 268.1080 (pink line), m/z 254.0924 (orange line), m/z 147.0552 (pink line) and at m/z 136.0757 (black line).

| *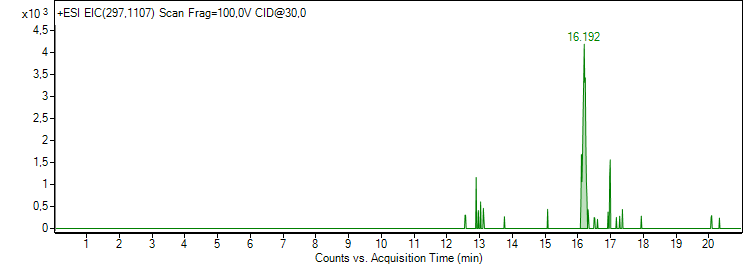*  *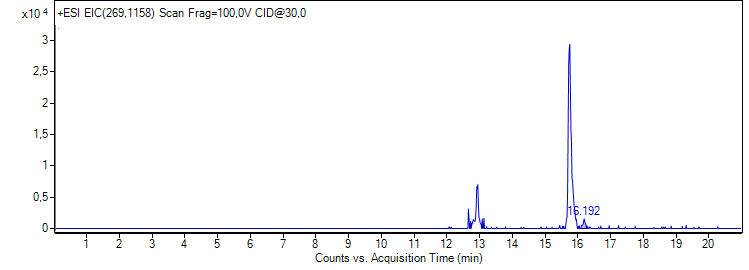*  *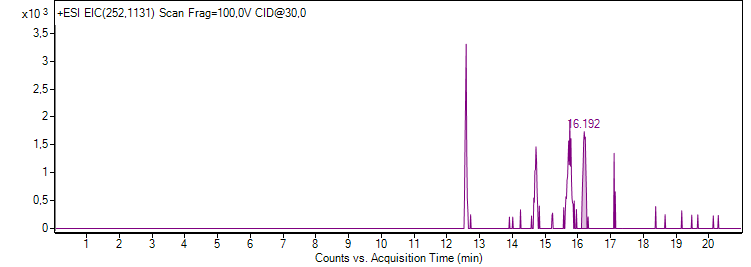* |
| --- |

## **Figure_SI-14_Omeprazole TP_12**, m/z 312.1342_ EIC of fragment at T28_EIC at m/z 297.1107 (green line), m/z 269.1158 (blue line) and at m/z 252.1131 (violet line).

**Spectra of Omeprazole TPs (2-12) at T28 (Figure SI-15-Figure SI-25).**

| 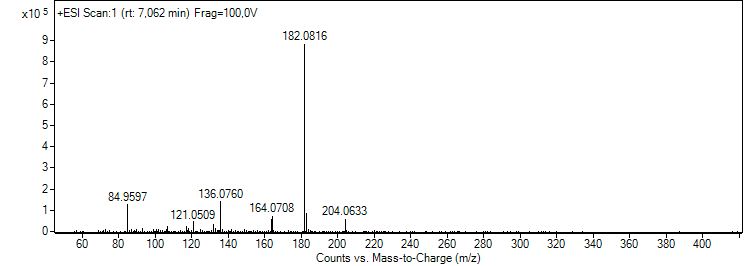 | 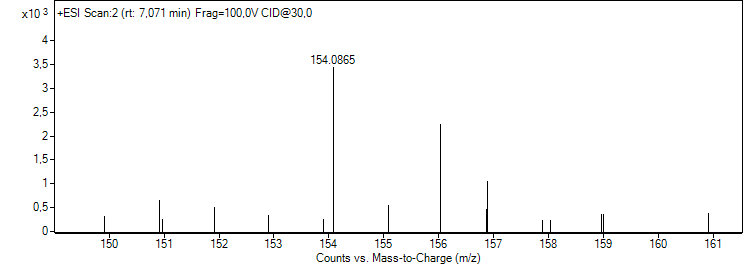  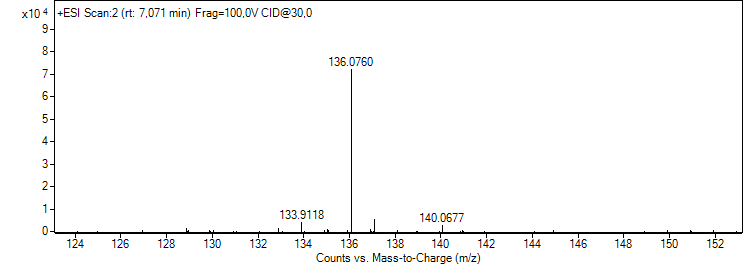 |
| --- | --- |

## **Figure SI-15** **Spectrum of Structure omeprazole TP_2**. Accurate mass spectrum at m/z 182.0816 and 7.062 min (left) then the corresponding MS/MS fragmentation pattern at 30eV (right) with ions at m/z 154.0865 and 136.0760.

| 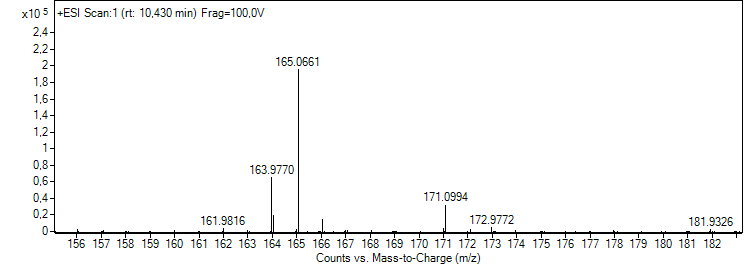 | 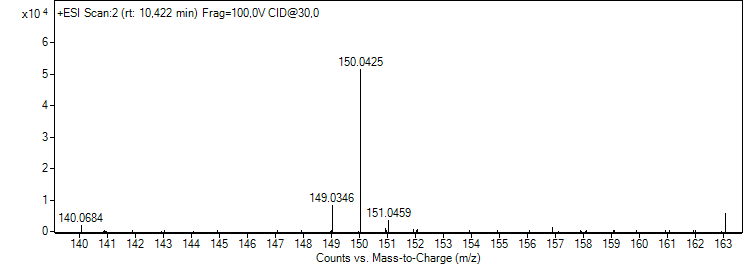  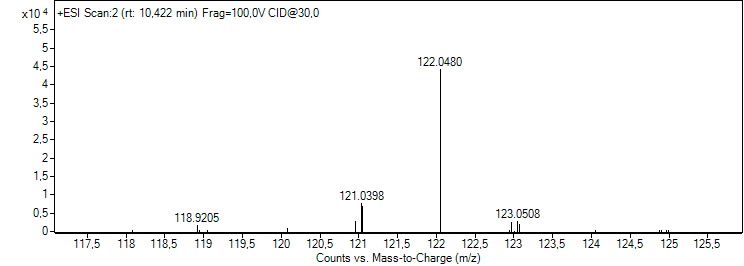 |
| --- | --- |

## **Figure SI-16** **Spectrum of Structure omeprazole TP_3**. Accurate mass spectrum at m/z 165.0661 and 10.430 min (left) then the corresponding MS/MS fragmentation pattern at 30eV (right) with ions at m/z 150.0425 and 122.0480.

| 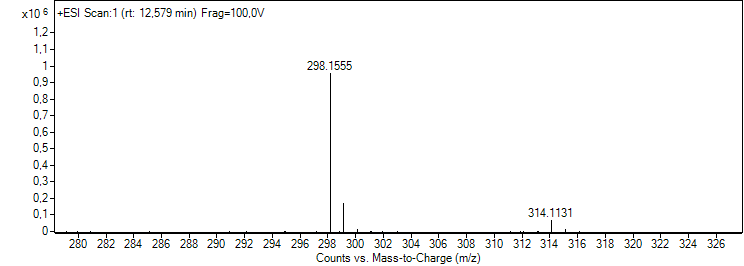 | 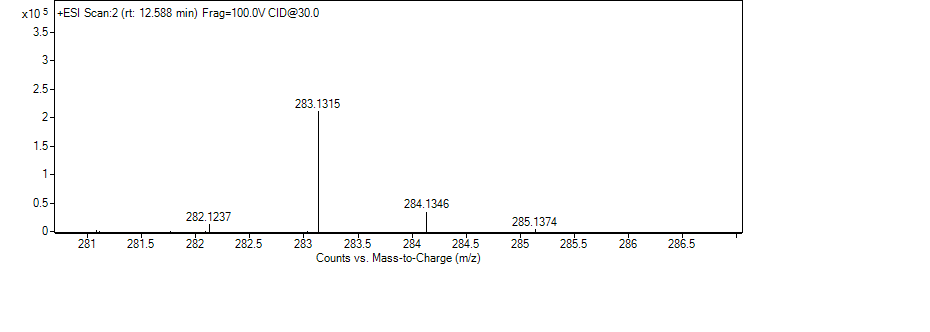  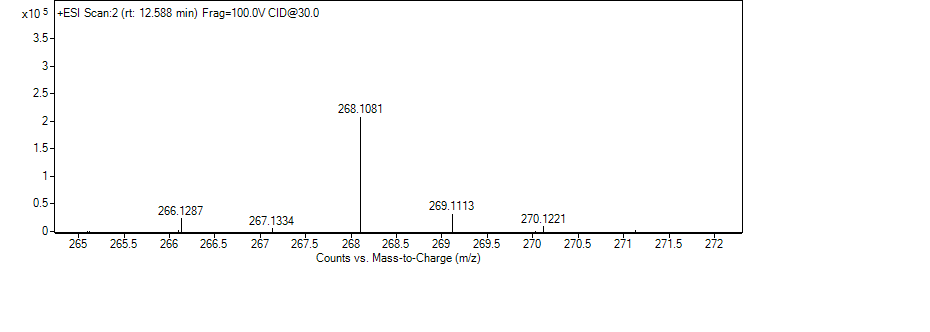  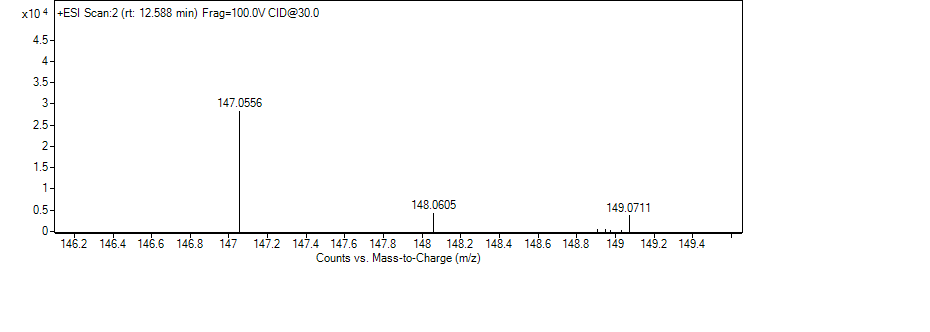  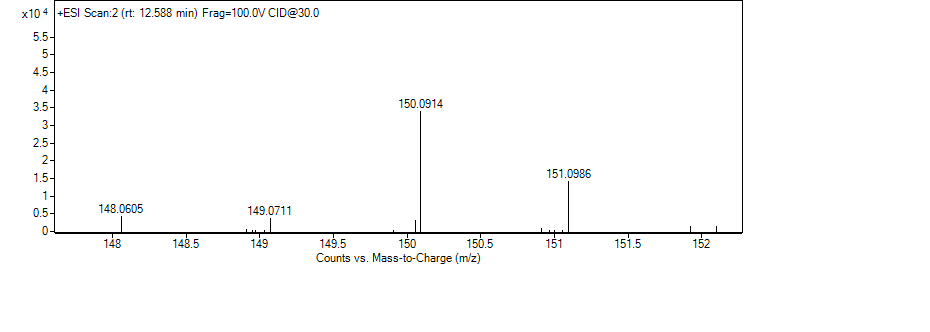  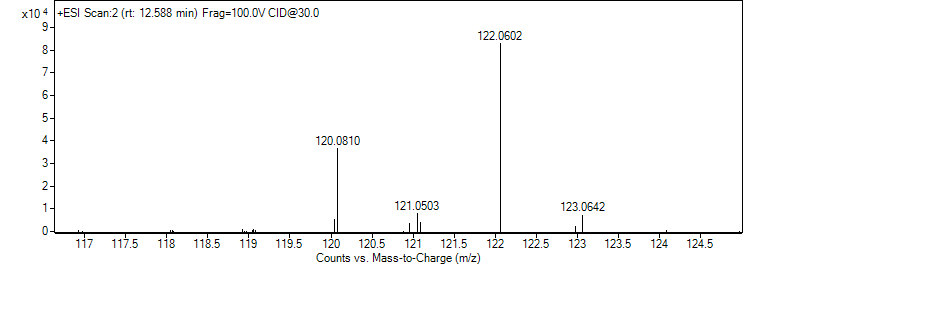 |
| --- | --- |

## **Figure SI-17** **Spectrum of Structure omeprazole TP_4.** Accurate mass spectrum at m/z 298.1555 and 12.579 min (left) then the corresponding MS/MS fragmentation pattern at 30eV (right) with ions at m/z 283.1315, 268.1081, 147.0556, 150.0914 and 120.0810.

| 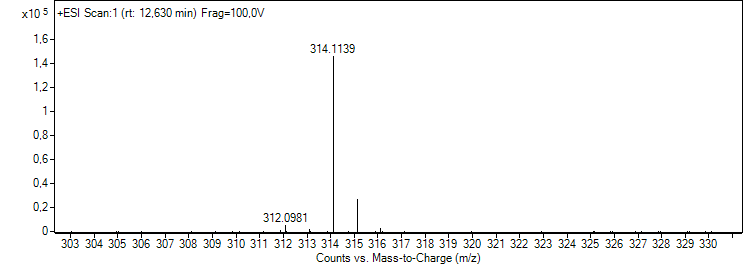 | 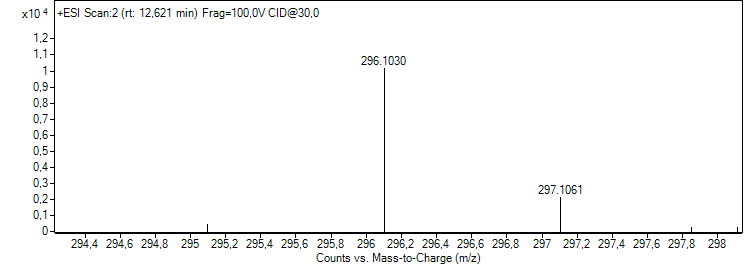  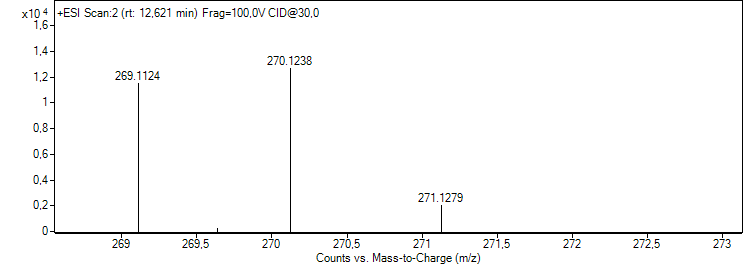  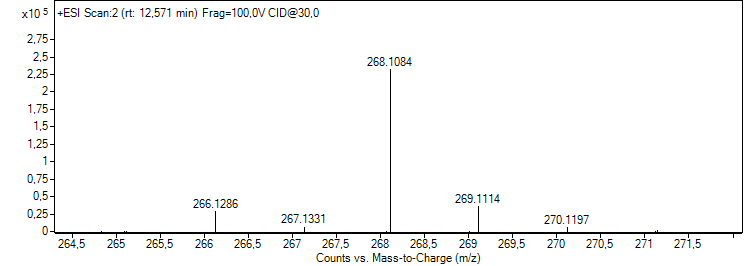  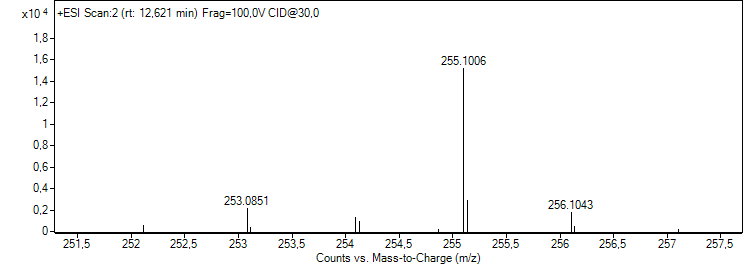  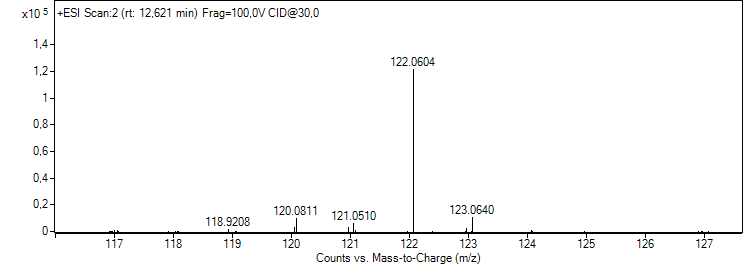 |
| --- | --- |

## **Figure SI-18** **Spectrum of Structure omeprazole TP_5.** Accurate mass spectrum at m/z 314.1139 and 12.630 and 12.839 min (left) then the corresponding MS/MS fragmentation pattern at 30eV (right) with ions at m/z 296.1030, 270.1238, 268.1084, 255.1006 and 122.0604.

| 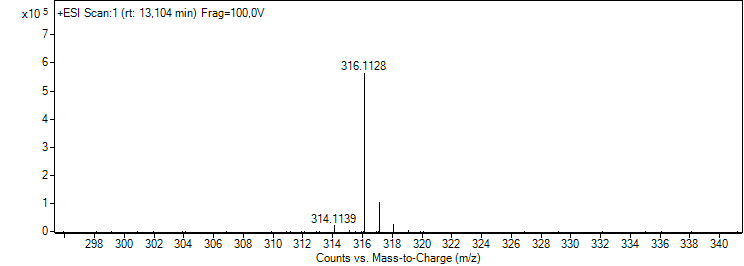 | 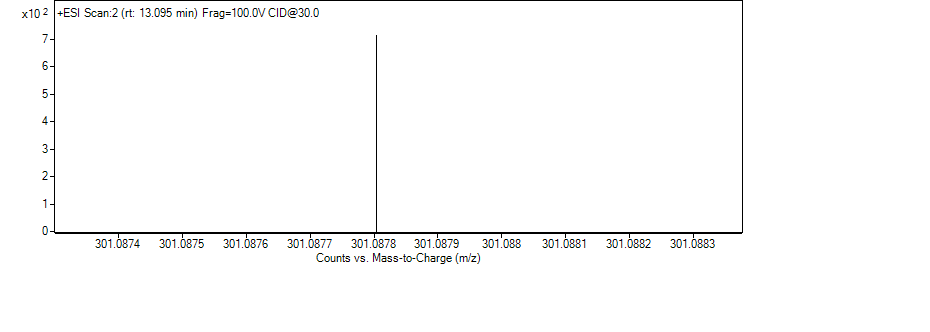  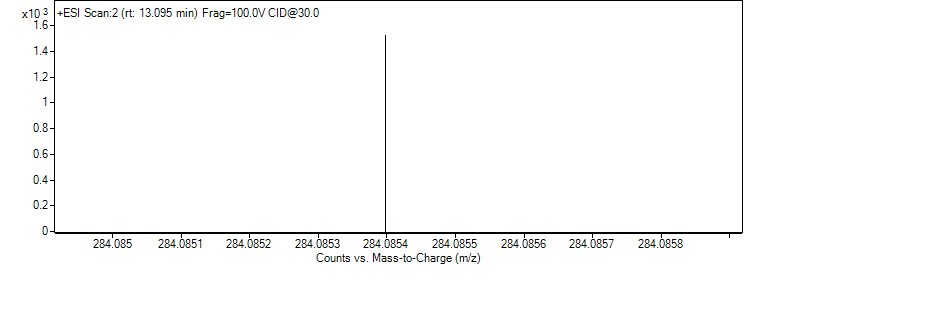  **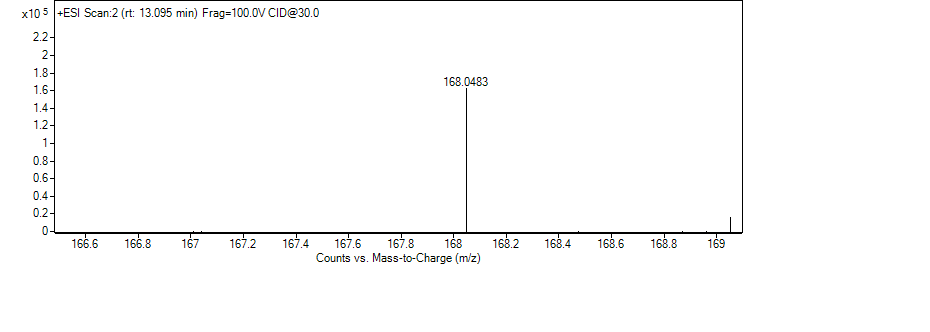**  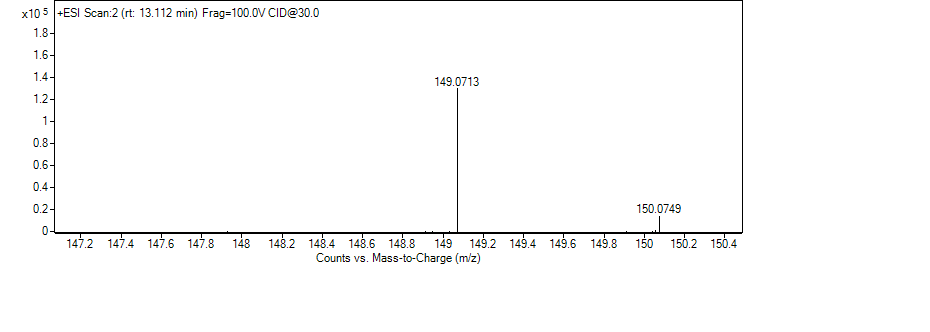  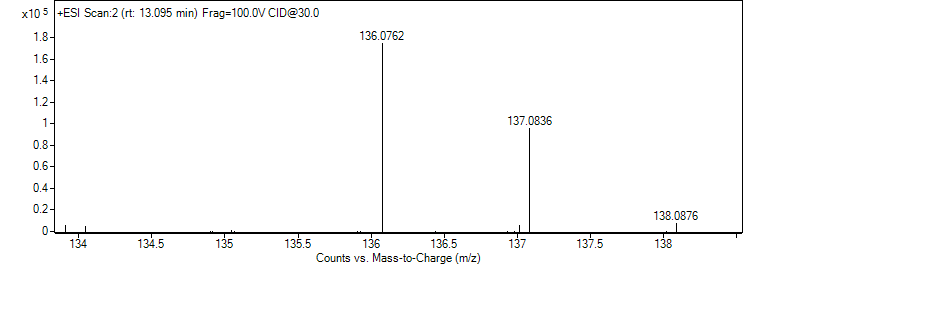 |
| --- | --- |

## **Figure SI-19** **Spectrum of Structure omeprazole TP_6.** Accurate mass spectrum at m/z 316.1128 and 13.104 min (left) then the corresponding MS/MS fragmentation pattern at 30eV (right) with ions at m/z 301.0878, 284.0853, 168.0483, 149.0713, 136.0762.

| 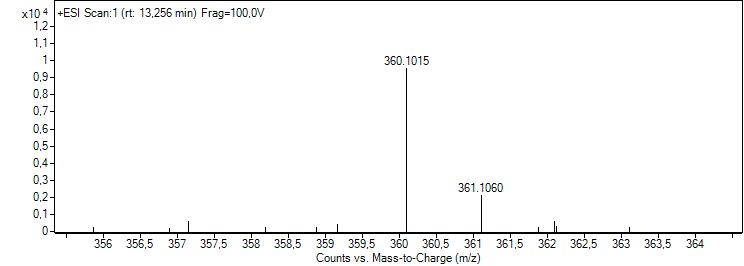 | 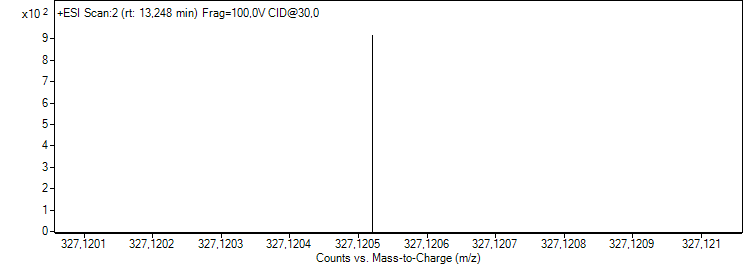  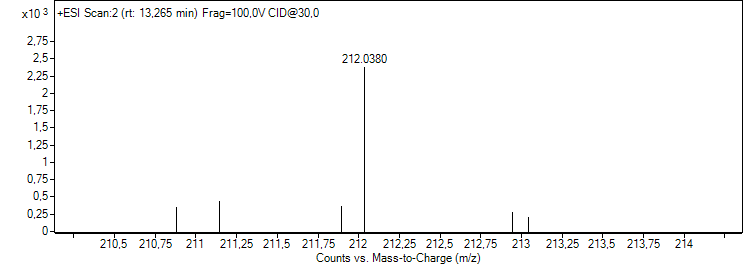  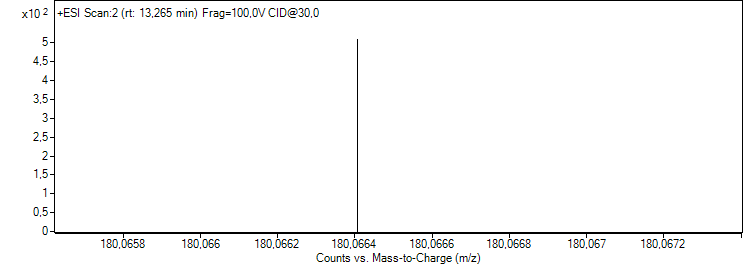 |
| --- | --- |

## **Figure SI-20** **Spectrum of Structure omeprazole TP_7.** Accurate mass spectrum at m/z 360.1015 and 13.227 min (left) then the corresponding MS/MS fragmentation pattern at 30eV (right) with ions at m/z 327.1205, 212.0380, 180.0664.

| 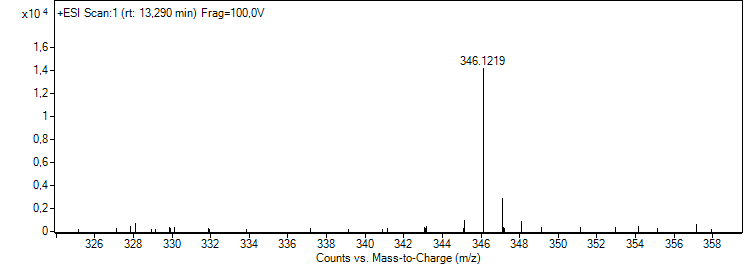 | 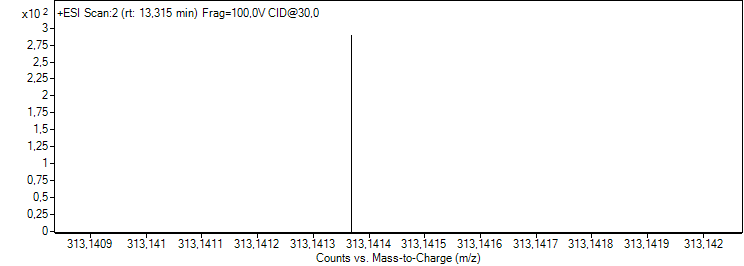  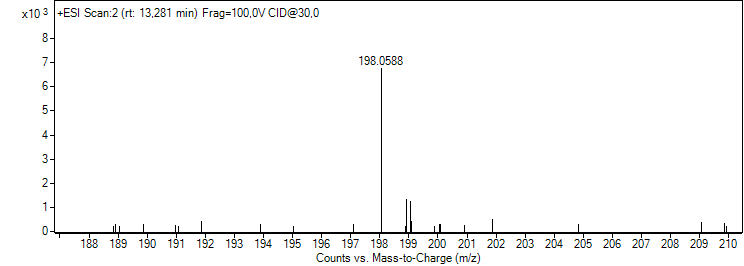  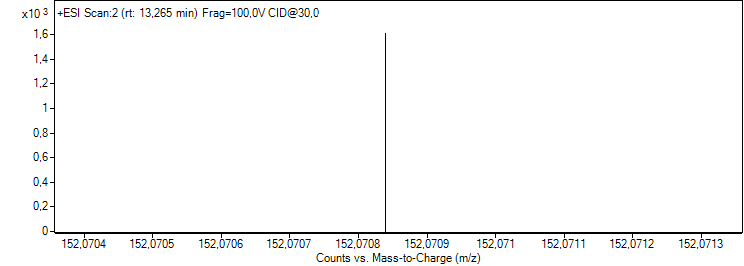  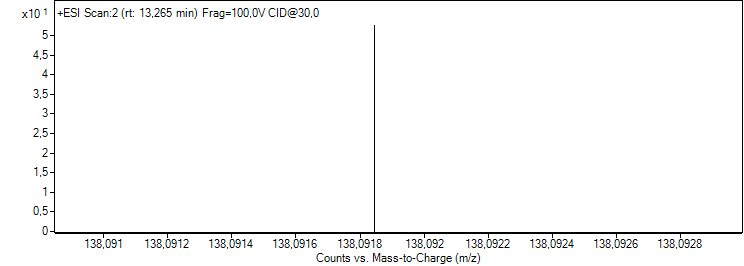 |
| --- | --- |

## **Figure SI-21** **Spectrum of Structure omeprazole TP_8.** Accurate mass spectrum at m/z 346.1219 and 13.290 min (left) then the corresponding MS/MS fragmentation pattern at 30eV (right) with ions at m/z 313.1414, 198.0588, 152.0708, 138.0918.

| 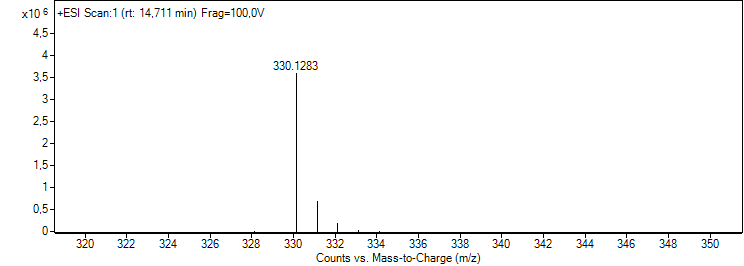 | 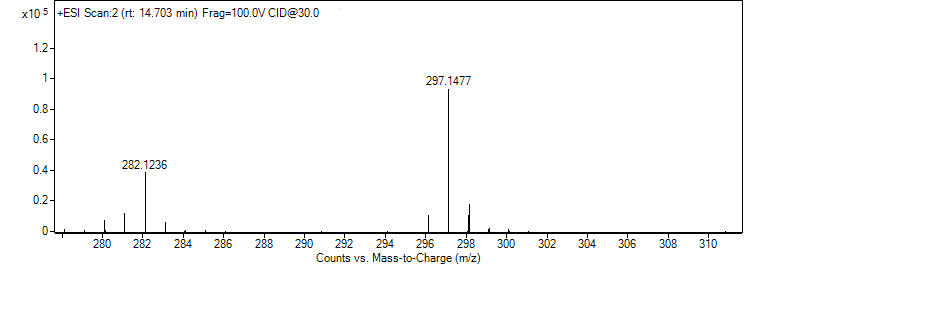  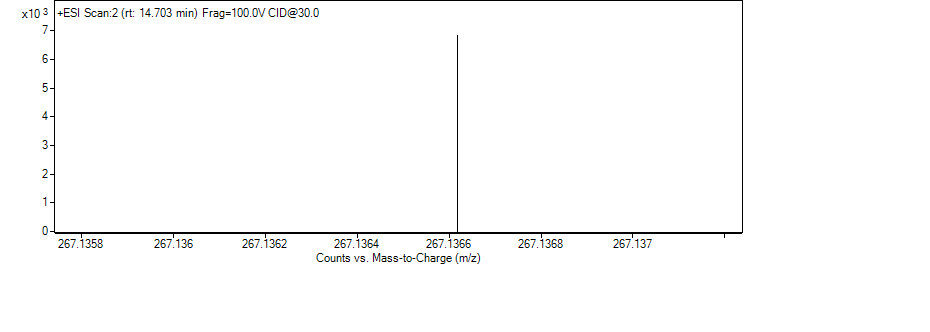  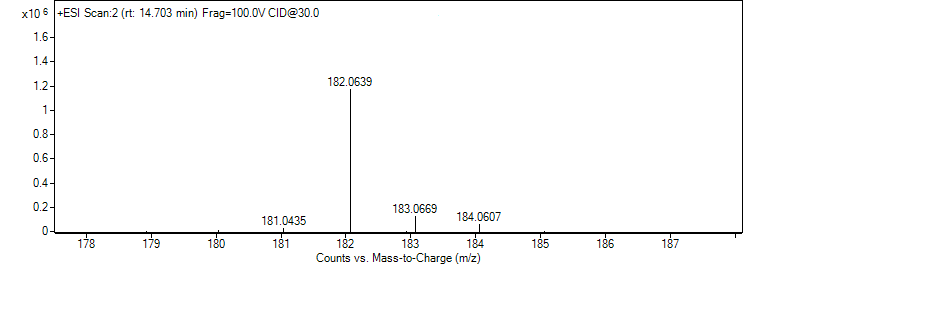  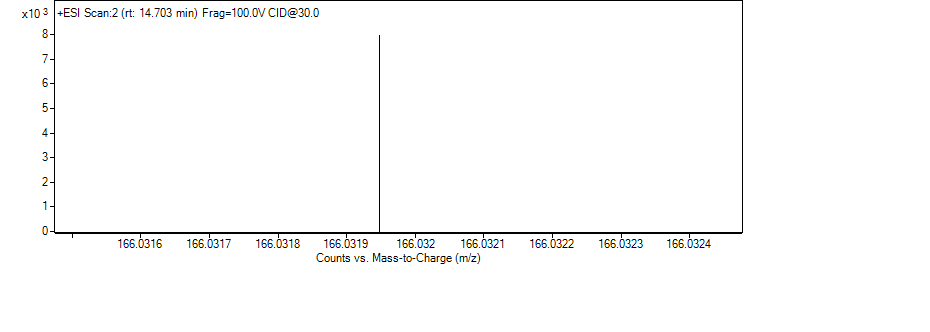  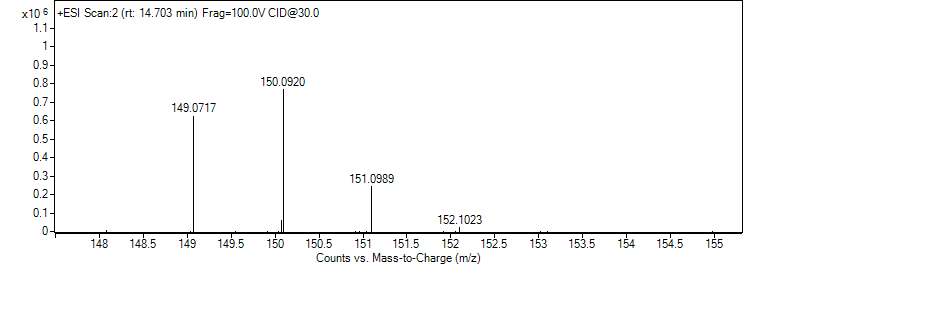  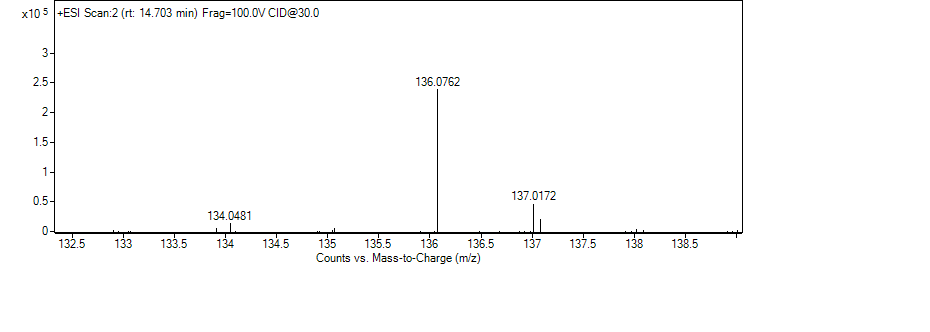  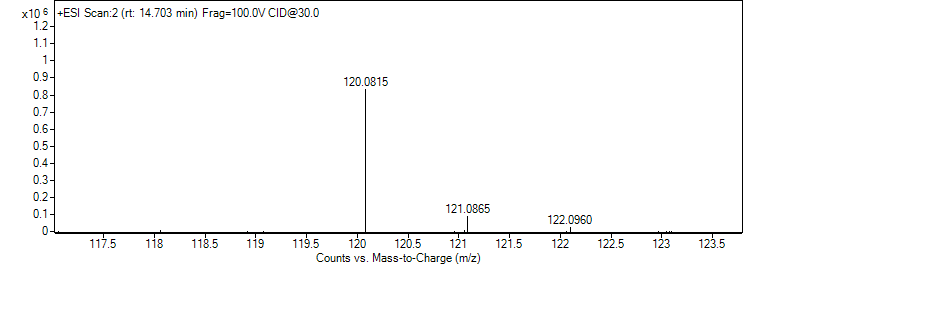 |
| --- | --- |

## **Figure SI-22** **Spectrum of Structure omeprazole TP_9.** Accurate mass spectrum at m/z 330.1283 and 14.711 min (left) then the corresponding MS/MS fragmentation pattern at 30eV (right) with ions at m/z 297.1477, 267.1366, 182.0639, 166.0320, 150.0920, 149.0717, 136.0762, 120.0815.

| 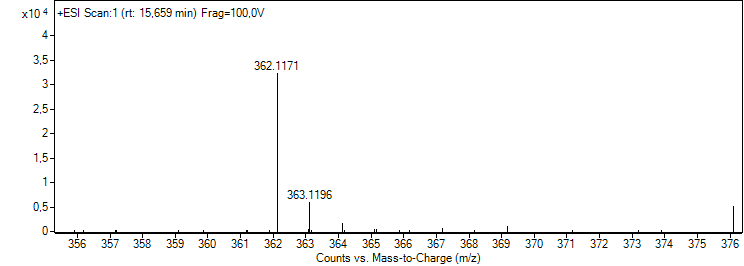 |  |
| --- | --- |

## **Figure SI-23** **Spectrum of Structure omeprazole TP_10.** Accurate mass spectrum at m/z 362.1171 and 15.659 min (left) then the corresponding MS/MS fragmentation pattern at 30eV (right) with ions at m/z 195.0218, 166.0872, 150.0917, 149.0718, 120.0811.

|  |  |
| --- | --- |

## **Figure SI-24** **Spectrum of Structure omeprazole TP_11.** Accurate mass spectrum at m/z 284.1397 and 15.744 min (left) then the corresponding MS/MS fragmentation pattern at 30eV (right) with ions at m/z 269.1163, 268.1076, 254.0930, 147.0555, 136.0716.

|  |  |
| --- | --- |

## **Figure SI-25** **Spectrum of Structure omeprazole TP_12.** Accurate mass spectrum at m/z 312.1352 and 16.218 min (left) then the corresponding MS/MS fragmentation pattern at 30eV (right) with ions at m/z 297.1107, 269.1161, 252.1126.
